# Evolution of Size‐Fecundity Relationship in Medaka Fish From Different Latitudes

**DOI:** 10.1111/mec.17578

**Published:** 2024-11-05

**Authors:** Shingo Fujimoto, Bayu K. A. Sumarto, Iki Murase, Daniel F. Mokodongan, Taijun Myosho, Mitsuharu Yagi, Satoshi Ansai, Jun Kitano, Satoshi Takeda, Kazunori Yamahira

**Affiliations:** ^1^ Integrated Technology Center University of the Ryukyus Okinawa Japan; ^2^ Tropical Biosphere Research Center University of the Ryukyus Okinawa Japan; ^3^ Laboratory of Molecular Reproductive Biology, Institute for Environmental Sciences University of Shizuoka Shizuoka Japan; ^4^ Graduate School of Fisheries and Environmental Sciences Nagasaki University Nagasaki Japan; ^5^ Department of Genomics and Evolutionary Biology, Ecological Genetics Laboratory National Institute of Genetics Mishima Japan; ^6^ Ushimado Marine Institute Okayama University Setouchi Japan; ^7^ Research Center for Marine Biology, Graduate School of Life Sciences Tohoku University Aomori Japan

**Keywords:** fecundity selection, life history adaptation, operational sex ratio, *Oryzias latipes* species complex, sexual selection

## Abstract

In most fishes, the number of offspring increases with maternal body size. Although this size‐fecundity relationship often varies among species as a result of the coevolution of life‐history traits, the genetic basis of such size‐fecundity relationships remains unclear. We explored the genetic basis underlying this size‐fecundity relationship in two small medaka species, *Oryzias latipes* and *O. sakaizumii*. Our findings showed that *O. sakaizumii* has a higher fecundity than *O. latipes*, and quantitative trait locus analysis using interspecific F_2_ hybrids showed that chromosome 23 is linked to the size‐fecundity relationship. In particular, the genes *igf1* and *lep‐b* in this region are known to be associated with life‐history traits, including somatic growth, gonad maturation, and progeny numbers in various taxa. Because *O. sakaizumii* is distributed at higher latitudes and has a shorter spawning season than *O. latipes* in the wild, we propose that the relatively high fecundity observed in *O. sakaizumii* is an adaptation to high latitudes. We also discuss the potential ecological ramifications associated with the evolution of increased fecundity in this species.

## 1 Introduction

In numerous taxa, the number of offspring per reproductive event, thats is, fecundity, increases with larger body size in a population, for example, reptiles (Shine 2005), insects (Berger, Walters, and Gotthard 2008; Green and Extavour 2014), and fishes (Barneche et al. 2018). This relationship between size and fecundity has been shown to vary markedly among species (Bunger et al. 2005; Orgogozo, Broman, and Stern 2006; Barneche et al. 2018; Healy et al. 2019; Álvarez‐Noriega et al. 2023). From an ontogenetic perspective, the amount of energy invested in reproduction changes throughout the development of an individual and is influenced by factors such as age and body size (Stearns 1992; Kooijman 2010; White et al. 2022). For example, ontogenetic changes in investment for reproduction are associated with physiological trade‐offs in energy allocation between survival, growth, and reproduction (Kooijman 2010; Simmons, Lupold, and Fitzpatrick 2017; Healy et al. 2019; White et al. 2022), all of which are subject to adaptive evolution in relation to life‐history traits (Stearns 1992; Simmons, Lupold, and Fitzpatrick 2017; White et al. 2022). Hence, the ecological factors and selection pressures shaping the size‐fecundity relationships within species have been extensively studied (Pianka 1960; Healy et al. 2019; White et al. 2022) to understand the coevolution of life‐history traits between species (Charnov, Turner, and Winemiller 2001; White et al. 2022; Álvarez‐Noriega et al. 2023).

Investigations into the genetic mechanisms that affect fecundity have focused primarily on intraspecific variation in domesticated animals that have been reared to maximize agricultural productivity (see review by Bunger et al. 2005). For example, both quantitative trait locus (QTL) analysis and genome‐wide association studies (GWAS) have been employed to elucidate the genetic basis of fecundity in chickens (Wright et al. 2010; Wolc et al. 2014; Zhao et al. 2021), quails (Minvielle et al. 2005), and salmonids (Sauvage et al. 2012; D'Ambrosio et al. 2020). Some genetic analyses have targeted wild populations; for example, the heritability of fecundity has been examined in the great tit, *Parus major* using genome‐wide nuclear genetic markers (Santure et al. 2013). In these studies, the heritability of fecundity ranged from low to moderate (0.14–0.42), as estimated by genome‐wide nuclear genetic markers (Santure et al. 2013; Wolc et al. 2014; D'Ambrosio et al. 2020). Single nucleotide polymorphisms (SNPs) contained in a single quantitative trait locus (QTL) explain only 1%–3% of the total genetic variance (e.g., D'Ambrosio et al. 2020). Although several candidate genes influencing intraspecific variation in fecundity have been identified (Wright et al. 2010; Swanson and Dantzer 2014; D'Ambrosio et al. 2020; Zhao et al. 2021), it remains unclear whether these genes can also explain interspecific variation in size‐fecundity relationships.

To explore the genetic basis of interspecific variation in size‐fecundity relationships, we focused on medaka, or ricefish, in genus *Oryzias*, which is widely used as an animal model in genetics and molecular biology (Iwamatsu 2006; Kinoshita et al. 2009). Medaka can be reared in the laboratory, which facilitates the genetic analysis of various phenotypes (e.g., Myosho et al. 2018; Ansai et al. 2021; Montenegro et al. 2022; Flury et al. 2022; Hilgers et al. 2022). In addition, medaka exhibit intraspecific variation in egg number (Leaf et al. 2011) and interspecific variation in spawning interval (Montenegro et al. 2022; Hilgers et al. 2022). In particular, the two species found in the Japanese archipelago (*Oryzias latipes* and *O. sakaizumii*) have been observed to spawn up to 50 eggs upon reaching sexual maturity. These two species are suitable for quantitative analysis of size‐fecundity relationships due to their similar adult size ranges, approximately 20–35 mm in standard length (SL; e.g., Awaji and Hanyu 1987; Kawajiri et al. 2014). A major empirical challenge in quantifying the size‐fecundity relationship in fishes is that fecundity generally increases exponentially with size (White et al. 2022), complicating the extrapolation and comparison of estimates between species with different size ranges. Thus, the similar adult size range of the two species makes them well‐suited to genetic analyses of reproductive traits. Although the two species are genetically distinct, with an estimated 2.45% *p*‐distance in genome‐wide nuclear single nucleotide variants between reference sequences (Ichikawa et al. 2017), they can be hybridized easily in captivity (Sakaizumi, Yasushi, and Satoshi 1992). Consequently, quantitative trait locus analysis in F_2_ individuals can be performed by using interspecific crosses (e.g., Tsuboko et al. 2014; Kawajiri et al. 2014, 2015; Yassumoto et al. 2020), which enables the analysis of the genetic basis of size‐fecundity relationships in the two species.

**FIGURE 1:**
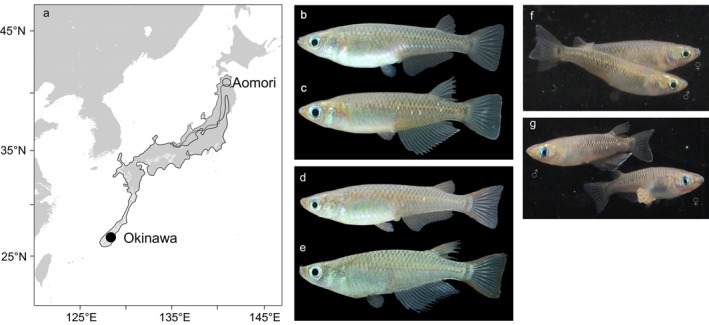
(a) Distribution range of *Oryzias latipes* (solid line) and *O. sakaizumii* (dotted line) in Japan (Takehana et al. 2003; Katsumura et al. 2019). Solid and open circles on the map represent the geographic locations of *O. latipes* and *O. sakaizumii* collection sites, respectively. Photographs of (b) *O. sakaizumii* female, (c) *O. sakaizumii* male, (d) *O. latipes* female, and (e) *O. latipes* male. (f, g) Egg spawning behavior during the mating sequence of *O. sakaizumii*. All photographs were taken by Shingo Fujimoto.

We hypothesize that adaptation to environmental conditions drives the evolution of the size‐fecundity relationships in the two species. *Oryzias latipes* and *O. sakaizumii* exhibit a parapatric distribution, in which *O. sakaizumii* tends to be distributed at higher latitudes than *O. latipes* in Japan (Figure 1). This distribution suggests that *O. sakaizumii* has evolved life‐history traits to cope with the harsher, more seasonal environments of higher latitudes. Common environment experiments have shown differences in growth, feeding activity, and maturation exist between these two medaka species (Yamahira and Takeshi 2008; Suzuki, Miyake, and Yamahira 2010; Fujimoto, Miyake, and Yamahira 2015; Shinomiya et al. 2023). Evidence obtained from seasonal fluctuations in gonad weight in wild populations (Awaji and Hanyu 1987; Fujimoto et al. 2021) combined with evidence of genetic variation in photo‐thermal responses to ovarian maturation (Shinomiya et al. 2023) suggests that constraints on physiologically suitable reproductive periods at elevated latitudes may impose severe selection pressures on life‐history traits in *O. sakaizumii* relative to *O. latipes*. Consequently, natural selection may favor relatively high fecundity in *O. sakaizumii* compared to *O. latipes* in order to optimize lifetime reproductive success within shorter reproductive seasons (e.g., Pincheira‐Donoso and Hunt 2017; Tarr et al. 2019).

However, no ecological studies have quantitatively compared the size‐fecundity relationships and spawning season duration, that is, the duration of female reproduction, between wild populations of *O. latipes* and *O. sakaizumii*. In this study, we examined these demographic characters to determine whether *O. sakaizumii* demonstrates a propensity to produce more eggs than *O. latipes* in their natural habitats. Information on the reproductive seasonality and fecundity of wild populations will provide insights into the adaptive evolution of life history and the selection pressures acting on these wild populations.

In addition, to clarify the contribution of genetic variation to fecundity in these species, common‐environment experiments were conducted using laboratory‐reared offspring. We also conducted QTL analysis using interspecific F_2_ hybrids to identify which chromosomal region is associated with interspecific variation in size‐fecundity relationships. These findings provide a promising foundation for elucidating the genetic and physiological mechanisms underlying the adaptive evolution of the size‐fecundity relationship.

## 2 Materials and Methods

### 2.1 Fish and Field Collection

Medaka, or the *Oryzias latipes* species complex, are small freshwater fish distributed in rice fields and low‐lying wetlands throughout Japan, Korea, and China (Iwamatsu 2006; Figure 1a). Molecular phylogenetic analyses have shown that the Japanese populations of the *O. latipes* species complex consist of two genetically distinct groups (Katsumura et al. 2019; Takehana et al. 2003, 2004); the ‘Northern Japan group’ or *O. sakaizumii*, which is distributed along the coast of the Sea of Japan (Asai, Senou, and Hosoya 2011; Figure 1b,c), and the ‘Southern Japan group’ or *O. latipes*, which is distributed along the coasts of the Pacific and the East China Sea (Figure 1d,e). The original map was generated using Generic Mapping Tools (Wessel et al. 2013).

In the laboratory, medaka reach sexual maturity at about 3 months of age (e.g., Kawajiri et al. 2014). The median life span of *O. latipes* under laboratory conditions is approximately 22 months (Ding et al. 2010). Both species have an ecological longevity of less than 2 years (e.g., Awaji and Hanyu 1987; Egami, Terao, and Iwao 1988; Fujimoto et al. 2021). Ovarian maturation is triggered by warmer temperatures and longer photoperiod (Koger et al. 1999; Shinomiya et al. 2023). Consequently, in the wild, the reproductive season is limited to the warm season, with gonad weight increasing from April to August (Awaji and Hanyu 1987; Fujimoto et al. 2021). During the reproductive season, individuals produce 10–30 eggs almost daily. Mating typically occurs between one male and one female in the water column (Figure 1f). After the female releases the eggs, the male fertilizes the eggs externally while they are attached to the female's genital pore (Figure 1g).

In this study, we collected individuals from two wild medaka populations: *O. sakaizumii* were collected from Aomori (40°50′N, 140°49′E) and *O. latipes* from Okinawa (26°49′N, 128°16′E) (Figure 1a). Aomori and Okinawa are located near the northern and southern distribution limits of *O. sakaizumii* and *O. latipes*, respectively (Iwamatsu 2006). Fish from each population were transferred to our laboratory, where they were housed in acrylic tanks for use as stock populations and for QTL analysis.

Throughout the laboratory rearing experiment described below, the water temperature of the tanks was maintained at 26°C (±0.1°C) and the photoperiod was maintained at 14 L:10D. Fish were fed daily on a diet consisting of newly hatched (24‐h‐old) nauplii of *Artemia franciscana* (Argent Chemical Laboratories, Redmond, WA, USA) and dry feed (Fancy Guppy, Hikari Tropical, Kyorin Corporation, Tokyo, Japan).

### 2.2 Spawning Season Assessment in the Wild

To examine spawning season and size‐fecundity relationships in the wild, we collected wild individuals from Aomori city (40°49′N, 140°48′E), and Kunigami village, Okinawa Island (26°49′N, 128°16′E) (Figure 1a). The collections in Aomori were conducted at 2‐week intervals between April and August in 2013. Similarly, collection in Okinawa were mainly conducted between March and October 2012. To confirm the non‐spawning season in Okinawa, additional collection was performed in December 2012 and February 2013. Moreover, to assess the possible role of two large typhoons that passed over Okinawa Island in 2012 on the termination of medaka spawning behavior, we conducted additional collections in Okinawa between August and October in 2013. We collected 62–1472 individuals per collection effort (Table S1). The collected fish were sexed based on sexually dimorphic characters (e.g., Kawajiri et al. 2014), and the adult sex ratio (adult males/adult males and females) in each collection was determined (Aomori: 0.44 ± 0.06, Okinawa: 0.51 ± 0.05, mean ± SD, Table S1).

Following collection, we randomly selected 20 adult females (i.e., > 20 mm SL to the nearest 1 mm using a ruler) and 20 adult males and transported them to a laboratory to evaluate seasonal changes in the proportion of sexually mature males and females. The following trials were repeated immediately after each field collection (Figure S1). To evaluate the reproductive ability of wild males and females, mating trials were conducted using either a single focal female or male paired with stock males and females, respectively. The stock individuals used in the mating trials consisted of wild males collected in Okinawa (verified in a preliminary survey) and laboratory‐reared offspring of females from Aomori (Figure S1). Using fertile stock individuals, we were able to independently assess the reproductive ability of wild individuals in both sexes.

We estimated the ratios of sexually mature individuals using a generalized additive model (GAM, Wood 2006). The GAM was estimated using the “gam” function in the “mgcv” package in the R program (Wood 2011). The outcome of the mating experiment for individual males (fertilization success/failure) and females (no clutch, one clutch, or two clutches) was treated as the response variable with a binomial distribution. We assumed a seasonal trend and fitted a smoothed curve between the collection dates, considering sex, population, and their interactions as explanatory variables. The significance of each term in the GAM was evaluated using a likelihood‐ratio test, with each term systematically dropped to assess its relative contribution (Tables S2 and S3). Upon confirming the significance of each term, we selected the best‐fitting model (Model 5, Table S3) to represent the seasonal variation in the proportion of mature individuals.

During the mating trial, the experimental fish were housed within a polypropylene container (160 × 120 × 80 mm) immersed into larger polypropylene tanks (900 × 620 × 210 mm, water depth 60 mm). The container had two holes (30 mm diameter) on each side and was covered with a dip‐net (150 × 125 × 70 mm, 32‐μm mesh), so that each pair spawned inside the dip‐net. Water in the tanks was circulated with a submersible pump and maintained at 26°C ± 1°C using immersion heaters. Each pair was allowed to mate freely, and the following morning (ca. 10:00 AM–12:00 PM), we checked for the presence of fertilized eggs, and fish were paired in this way for two consecutive days. After the mating trials, each wild fish was photographed and SL was measured based on these photographs (Optio x90, RICOH, Tokyo, Japan). The wild fish were then released at the collection site. Considering the habitat area and the population density, some of the individuals may have been recaptured and measured.

For the statistical analysis of size‐fecundity relationships in wild individuals, we used data from 71 spawned females from Aomori and 188 spawned females from Okinawa. We constructed a linear model to compare the size‐fecundity relationship between the two populations. Egg number was treated as the response variable, while female SL, population, and their interaction were treated as the explanatory variables. The significance of the explanatory variables was estimated using an *F* test. All statistical analyses were performed in R ver. 4.1.1 (R Core Team 2021). The linear model analysis was conducted using the “lm” function implemented in the MASS package. Type 3 ANOVA was conducted employing the “Anova” function in the “car” package (Fox and Weisberg 2019).

### 2.3 Establishment of F_2_ Hybrids for QTL Analysis

We reared F_2_ hybrid individuals from previous studies (Fujimoto et al. 2014; Kawajiri et al. 2014, 2015), which we used for QTL mapping in this study. In May 2011, we collected 34 adults from Aomori and 50 from Okinawa, with a sex ratio of approximately 1:1. We then established two F_2_ progeny lines: F_2_ progeny from F_1_ hybrids between an Aomori female and an Okinawa male (AFOM), and F_2_ progeny from F_1_ hybrids between an Okinawa female and an Aomori male (OFAM) (Figure S1). One male from one population and one female from the other population were randomly selected from the wild fish stock kept in the laboratory. The pair was maintained in a small polypropylene container (15 × 11 × 8 cm) placed within an acrylic tank (75 × 60 × 45 cm). Fertilized eggs were obtained from the mated pair and the resulting F_1_ individuals were transferred to small acrylic containers (25 × 20 × 23 cm) immersed in the larger acrylic tanks. These F_1_ individuals were then reared in the same containers. Following maturation, the F_1_ individuals were allowed to mate freely, and newly fertilized eggs of F_2_ individuals were collected.

### 2.4 Phenotypic Measurements

We performed mating experiments to measure the egg numbers and mating behaviors in F_2_ individuals for QTL analysis. The sex of each F_2_ individual was determined at 124 days post‐fertilization, after which each F_2_ individual was assigned to the mating trials described below. In total, we analyzed 92 OFAM females, 78 OFAM males, 87 AFOM females, and 101 AFOM males. F_2_ females were mated with wild stock males from Okinawa and F_2_ males were mated with laboratory‐reared offspring females from Aomori. These stock individuals were the same as those used in the mating trials with wild populations (Figure S1).

One day before each mating trial, we randomly selected one mating partner from the stock fish in the laboratory. The F_2_ individual and a mating partner were placed in a small acrylic tank (14 × 7.5 × 15 cm, 12‐cm water depth), which was immersed in a temperature‐controlled (26°C) acrylic tank (75 × 60 × 45 cm). The female and male were separated by an opaque partition. The following morning at approximately 9:30–10:00 AM, the trial was initiated by removing the partition, and the male and female were allowed to mate freely in the tank. We recorded their behavior for 1 h using a digital video camera (HDR‐CX180, Sony Corporation, Tokyo, Japan). After the trial was completed, the number of eggs was counted for the F_2_ females at approximately 17:00–18:00 PM in the evening. The mating trial was repeated twice for each F_2_ female and male for 2 days in succession. In the event that the F_2_ female spawned clutches on both trial days, the average number of eggs was calculated. As a result, we obtained egg number data for 65 OFAM females and 60 AFOM females.

In medaka fishes, factors such as age, nutrient condition, and ambient temperature have a marked effect on egg production (e.g., Leaf et al. 2011; Hemmer‐Brepson et al. 2014). To exclude these environmental and demographic factors between wild populations, a common environment experiment was conducted, and the egg numbers of laboratory‐reared offspring females were measured in 2021. The number of eggs produced by 17 Okinawa females and 21 Aomori females was measured in a manner similar to that described for F_2_ individuals.

We also analyzed the mating behaviors of the F_2_ females using the video recordings. In addition to the number of courtship behaviors observed until spawning, the time interval between the time of first courtship and successful egg spawning was recorded as spawning latency, as this is an indicator of female mate preference in medaka (e.g., Fujimoto et al. 2014; Ogino et al. 2023). Wrapping rejections (i.e., when a female disengages from male wrapping without releasing eggs) were also counted and used as an index of female mate preference. In the mating experiment of the F_2_ females, we used wild Okinawa males as mating partners.

The mating behaviors of F_2_ males were also examined. To evaluate the sexual motivation of F_2_ males, courtship latency, that is, the time interval between the removal of the separator and the initiation of courtship, frequency of approaches, and frequency of quick circles, that is, a male courtship behavior in which males approach females from below and then swim rapidly in a circle around the female (Ono and Uematsu 1957), were measured. For F_2_ male phenotypic measurements, we used laboratory‐reared Aomori females as mating partners. F_2_ males of pairs that did not spawn within 1 h were not used for subsequent QTL analysis, because male sexual motivation may have been affected by the reproductive condition of the mating partner (e.g., experimental females may not have produced mature eggs). We obtained courtship behaviors for 52 OFAM males and 80 AFOM males. These behavioral data of F_2_ males were originally published in Fujimoto et al. (2014).

### 2.5 Genotyping and QTL Mapping

For genotyping the F_2_ progeny, we used a custom VeraCode GoldenGate Genotyping Assay with 384 SNP panels (Illumina, San Diego, CA, USA) for Japanese medaka (Kawajiri et al. 2014). The linkage map of the OFAM family and the AFOM family was constructed in a previous study (Kawajiri et al. 2014, 2015). To assess the relationship between QTL for the number of eggs laid and observations of mating behavior, we employed multiple QTL mapping with the R/qtl package (Broman and Sen 2009; Arends et al. 2010). The false discovery rate (FDR) of logarithm of the odds (LOD) scores among peaks in multiple chromosomes were determined with 1000 bootstrap permutations (significant: FDR < 0.05, suggestive: 0.05 < FDR < 0.10). R/qtl was also used to compute 95% Bayesian credible intervals (95% BI) for QTL peak positions. QTL analyses for each phenotype were performed separately, with datasets treated by family and sex (Table S4).

In addition, the proportion of variance explained (PVE) for each phenotype was calculated to estimate the genetic variance (Table S4). PVE was calculated in three ways: (a) an estimate based on the covariance matrix between individuals using all genome‐wide genetic markers, (b) an estimate using only markers on the chromosome where the QTL peak was detected, and (c) an estimate using the genetic marker closest to the QTL peak. Genome‐wide PVE was calculated using the “mmer” function implemented in the “sommer” package (Covarrubias‐Pazaran 2016). To minimize the risk of false positives in the QTL analysis, phenotypes with low genetic variance (genome‐wide PVE < 1%) were excluded from the results, even if QTL peaks were detected (Table S4).

We investigated whether the QTL for egg number affected the allometric relationship between body size and egg number. The nearest genetic marker to the peak LOD score was selected for QTL mapping of egg number. Subsequently, a linear model was employed, followed by an ANOVA to assess the significance of the genetic marker, body size, and their interaction. A type 3 ANOVA was executed using the “Anova” function in the “car” package to mitigate compounding effects arising from the order of the terms (Fox and Weisberg 2019).

### 2.6 Gene Ontology Analysis

To explore the functional characteristics of genes located within the identified QTLs, we conducted gene ontology (GO) analysis. Gene names and GO terms annotations were downloaded from the Ensembl release 109 (https://feb2023.archive.ensembl.org/) for the *O. latipes* HdrR genome database (ASM223467v1, Ichikawa et al. 2017). Given that the reference genome of the HdrR strain in *O. latipes* (ASM223467v1) was better annotated than the HNI strain in *O. sakaizumii* (ASM223471v1), we chose to use the HdrR genome for our analysis. For example, the HNI strain lacks an extensive ortholog database, and gene symbols were undetermined in for most Ensembl gene IDs, whereas this information is available for the HdrR strain of *O. latipes*. We performed GO enrichment analysis using gene annotation information from the HdrR genome and ShinyGO ver. 0.80 (Ge, Jung, and Yao 2020). Enrichment analysis was performed separately for Ensembl gene IDs located within each QTL. Statistical significance of the enrichment was determined as FDR < 10^−5^. We examined the top 10 GO terms ranked by the FDR of fold enrichment. In addition, we also identified genes associated with GO terms such as “reproduction”, “oogenesis”, or “behavior” using a custom script and the “biomaRt” package (Durinck et al. 2005).

The original genetic markers used for QTL analysis (Kawajiri et al. 2014, 2015) were based on an earlier version of the reference genome of the HdrR strain in *O. latipes* (ASM31367v1, Kasahara et al. 2007). Subsequently, the genomic positions of these genetic markers were lifted from the older reference (ASM31367v1) to the newer version (ASM223467v1) using the LiftOver software package (Hinrichs et al. 2006); we performed this liftover using “flo” (https://github.com/wurmlab/flo/, Pracana et al. 2017), a pipeline that employs the UCSC tools (Kent 2002).

## 3 Results

### 3.1 Interspecific Variation in Fecundity

**FIGURE 2:**
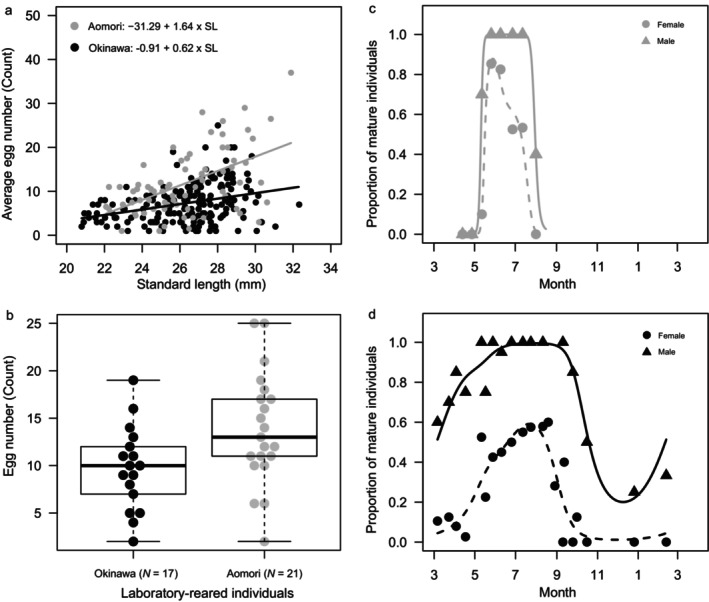
(a) Size‐fecundity relationship of *Oryzias latipes* (Okinawa), and *O. sakaizumii* (Aomori) females in the wild. (b) Average egg number in laboratory‐reared offspring females from Okinawa and in Aomori. (c) Seasonal change in the proportion of sexually mature individuals during 2‐day observation period in Aomori and (d) in Okinawa (circles: females, triangles: males). Solid and dashed lines (estimated using a generalized additive model) represent males and females, respectively.

Compared to the wild Okinawa population, Aomori females tended to produce more eggs in each clutch (Figure 2a). The results of the linear model analysis showed that the SL of females was positively correlated with the average number of eggs per clutch (SL; Estimate ± SE = 1.13 ± 0.15, *df* = 1, *F* = 59.58, *p* < 0.0001), and that the Aomori population tended to produce more eggs than the Okinawa population (population; Estimate ± SE = 11.1 ± 3.87, *df* = 1, *F* = 8.28, *p* = 0.004). Similarly, the interaction between SL and population was also significant (population × SL; Estimate ± SE = −0.51 ± 0.15, *df* = 1, *F* = 12.14, *p* = 0.0006), suggesting that the increase in the number of eggs produced by females from Aomori increased at a higher rate with body size compared to females from Okinawa (Figure 2a).

A significantly larger proportion of females from Aomori spawned two clutches compared to females from Okinawa (Fisher's exact test, *p* < 0.001, Figure S2); approximately 40% of females from Aomori produced two clutches during the two consecutive days of the experiment, but only 10% of Okinawa females produced two clutches (Figure S2). The results showed that the Aomori population has a higher fecundity than the Okinawa population in the wild.

A difference in the average number of eggs was also observed in lab‐reared females (Figure 2b), indicating that differences in average egg number observed between wild populations persisted when environmental and demographic factors were excluded. When SL was considered in the linear model, the variance in egg number was observed between Aomori and Okinawa (Estimate ± SE = 42.14 ± 15.48, *df* = 1, *F* = 7.41, *p* = 0.0102). In this experiment, laboratory reared offspring females did not show a significant difference in the mean SL between females from Aomori and Okinawa (*t* = −1.56, *df* = 32.20, *p* = 0.1286). The mean SL was 25.0 ± 1.2 mm in the Aomori population (*N* = 17) and 24.4 ± 1.4 mm in the Okinawa population (mean ± SD, *N* = 21). The trend in female SL was consistently observed in wild populations assessed for reproductive ability (*t* = −0.46, *df* = 237.3, *p* = 0.65). The mean SL was 25.2 ± 3.0 mm in the Aomori population (*N* = 157) and 24.4 ± 1.4 mm was in the Okinawa population (*N* = 437).

### 3.2 Spawning Season in the Wild

The spawning season of the Aomori population was shorter than the Okinawa population (Figure 2c,d; Table S2). Wild fish from Aomori spawned from May to July, whereas those in Okinawa spawned from March to October (Figure 2c,d; Table S2). A higher proportion of females matured during the peak of the spawning season in the Aomori population (June, 80%) compared to the Okinawa population (May–September, 50%–60%) (Figure 2c,d). The seasonal trend in the proportion of mature individuals differed significantly, not only between populations (Table S2, Model 2 vs. Model 3: *df* = 5.3, *deviance* = 141.0, *p* < 0.0001), but also between sexes and their interactions (Model 3 vs. Model 4: *df* = 9.4, *deviance* = 92.7, *p* < 0.0001; Model 4 vs. Model 5: *df* = 1.2, *deviance* = 283.0, *p* < 0.0001). Almost all of the males from both populations were sexually mature at the peak of the female spawning season (Figure 2c,d).

### 3.3 QTLs Associated With Phenotype

**FIGURE 3:**
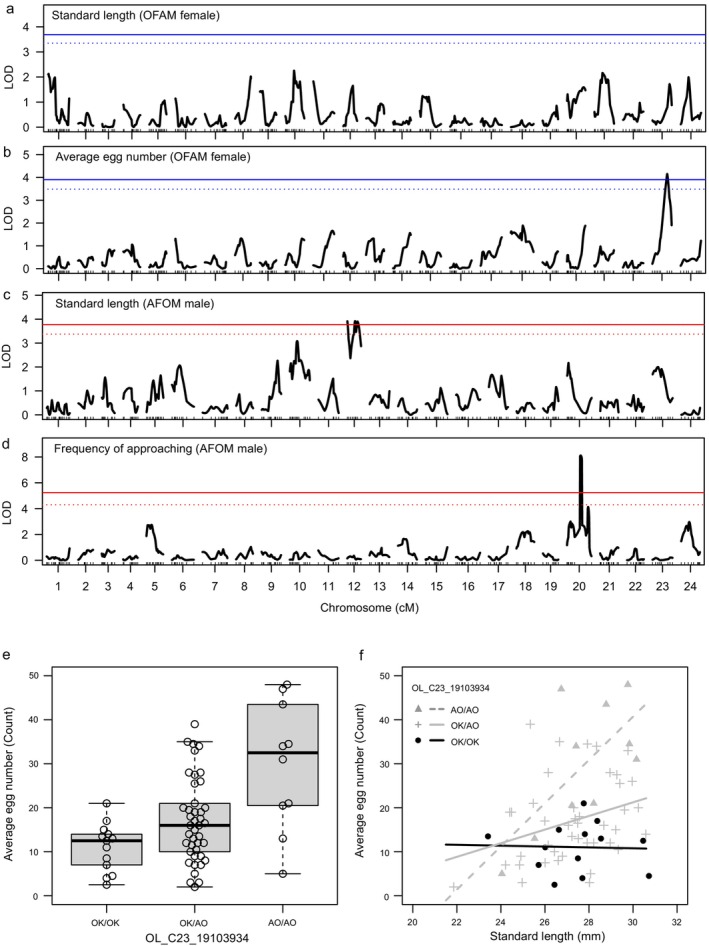
Quantitative trait locus analysis of the F_2_ hybrids between Okinawa and Aomori. (a, c) Logarithm of the odds (LOD) score for the standard length, (b) average egg number, (d) frequency of approaching. Lines show significance levels of false discovery rate (FDR) by genome‐wide permutation tests with multiple peaks (solid line: FDR < 0.05, dotted line: 0.05 < FDR < 0.10). (e) Average egg number for genotypes of single nucleotide polymorphism (SNP) marker OL_C23_19103934, which had the highest LOD score among the markers. (f) Size‐fecundity relationship for genotypes of the SNP marker OL_C23_19103934. Strain refers to F_2_ progeny derived from F_1_ hybrids between an Aomori female and an Okinawa male (AFOM, red lines), or F_2_ progeny derived from F_1_ hybrids between an Okinawa female and an Aomori male (OFAM, blue lines).

Six QTLs showed associations with the observed phenotypes (Table 1; Table S4) and three QTLs showed statistically significant (Table 1; Figure 3a–d). No significant and suggestive QTLs were shared between the AFOM and OFAM strains (Figure 3a–d; Table 1; Table S4; Figure S3). The QTLs associated with egg number were located on chromosome 23 in the OFAM strain (Table 1; Figure 3b). Analysis revealed that the average egg number was higher in alleles of the Aomori genotype compared to those of Okinawa at the nearest genetic marker of the peak LOD score (Figure 3e,f).

**TABLE 1:** Significant quantitative trait locus (QTL) (FDR < 0.05 by genome‐wide permutation tests) and suggestive QTL (0.05 < FDR < 0.10) associated with standard length, average egg number, spawning latency, wrapping rejection, and frequencies of approaching. Strain refers to F_2_ progeny derived from F_1_ hybrids between an Aomori female and an Okinawa male (AFOM), or F_2_ progeny derived from F_1_ hybrids between an Okinawa female and an Aomori male (OFAM).

					QTL candidate regions							HdrR, ASM223467v1	
Phenotype	Strain	*N*	Sex	Genome‐wide PVE	Chr	Pos (cM)	95% CI (cM)	LOD	Marker PVE	Chr PVE	FDR	95% CI (bp)	Coding sequences
Standard length	AFOM	101	M	48.2	12	23	0–38	3.92	16.3	33.8	*	7.81–27.84	296
Average egg number	OFAM	65	F	16.2	23	40.9	33–48	4.15	25.5	45.6	*	15.49–20.23	62
Spawning latency	OFAM	46	F	61.9	13	23	16–27	4.33	23.2	50.5	^a^	10.44–16.24	104
Spawning latency	OFAM	46	F		18	13	2–25	4.26	22.4	52.8	^a^	4.74–14.26	112
Wrapping rejection	OFAM	47	F	17.2	24	35	29–43	5.29	32.3	65.8	^a^	10.73–15.89	102
Frequency of approaching	AFOM	80	M	18.8	20	39	38–43	8.11	10.4	32.7	*	20.93–21.45	32

Abbrveiations: 95% CI: 95% Bayesean credible intervals; bp: 10^6^ base pair; Coding sequences; number of genes in the 95% CI QTL region; Chr: chromosome; cM: Centi Morgan; LOD: Logalithm of the odds; FDR: false discovery rate; PVE: phenotypic variance explained.

^a^ 0.05 < FDR < 0.10.

* FDR < 0.05.

Furthermore, we also assessed the influence of QTLs on the relationship between size and fecundity using a linear model (*F*_*5,59*_ = 8.11, adj *R* ^*2*^ = 0.36, *p* = 7.07e^−06^). The results showed that egg number increased in individuals with larger body size possessing the Aomori homozygous genotype (*N* = 10, slope = 4.92, intercept = −106.73) and heterozygous genotype (*N* = 42, slope = 1.55, intercept = −25.25), whereas the slope of egg number was significantly smaller in individuals with the Okinawa homozygous genotype (*N* = 13, slope = −0.10, intercept = 13.75) (Figure 3f). In the linear model, the main effect of SL was statistically significant (*df* = 1, *F*_1,59_ = 8.83, *p* = 0.004) and the interaction between SL and genotype was also significant (*df* = 2, *F* = 3.20, *p* = 0.048), whereas the main effect of genotype was not statistically significant (*df* = 2, *F*_2,59_ = 2.41, *p* = 0.099). To confirm that the detected egg number QTL had a statistically significant effect on the size‐fecundity relationship, we also examined the distribution of slope differences in the size‐fecundity relationship using all genetic markers. Two genetic markers (OL_23_19103934, OL_23_20828757) located within the egg number QTL were among the top 5% (13/273 markers) in the distribution, supporting the finding that the egg number QTL on chromosome 23 affected the size‐fecundity relationship.

Suggestive QTLs related to the female behaviors, specifically spawning latency and wrapping rejection, were identified on chromosomes 13, 18, and 24 (Table 1, Table S4). For both QTL 13 and 18, individuals with Aomori alleles tended to take longer to spawn than those with the Okinawa alleles (Figure S4e,f). Similarly, QTL 24, individuals with Aomori alleles exhibited a higher frequency of rejection behavior compared to those with Okinawa alleles (Figure S4c). In AFOM males, a significant QTL associated with SL was mapped to chromosome 12 (Table 1, Figure 3a). The frequency of approaching, which are indicator of male sexual motivation, demonstrated an association with chromosome 20 (Table 1, Figure 3d). Individuals with Aomori alleles exhibited a higher frequency of courtship behavior compared to those with Okinawa alleles (Figure S4b).

We performed GO enrichment analysis for each of the six QTLs (Table 1; Table S5). The significant enrichment was observed QTL of wrapping rejection in chromosome 24, including the term “Chemokine binding” (Table S5, Fold Enrichment = 52.97, FDR < 1e^−5^). No significant enrichment was observed for the other QTLs (Table S5). The GO terms with the lowest enrichment FDR for each QTL are listed below: for the QTL associated with SL, “response to heat” (Fold Enrichment = 15.64, FDR = 0.0023); spawning latency in chromosome 13, “G protein‐coupled receptor activity” (Fold Enrichment = 4.68, FDR = 0.0019); spawning latency in chromosome 18, “microtubule associated complex” (Fold Enrichment = 9.94, FDR = 0.1784); courtship frequency, “regulation of B cell proliferation” (Fold Enrichment = 271.26, FDR = 0.0025); and egg number, “glycoprotein metabolic process” (Fold Enrichment = 9.44, FDR = 0.0475).

In addition, to identify the candidate genes within the three significant QTL regions falling within the 95% Bayesian credible intervals (Table 1), we conducted a search for genes associated with “reproduction”, “oogenesis”, and “behavior” utilizing the GO database (ASM223467v1, Ensembl 109, https://feb2023.archive.ensembl.org). QTLs located on chromosomes 20 and 23 were found to harbor three genes related to “reproduction”, namely THAP Domain Containing 6 (*THAP6*), and insulin‐like growth factor 1 gene (*igf1*) (Table S6). Notably, the QTL region on chromosome 23 contained *igf1*, as well as the leptin gene (*lep‐b*), a gene potentially related to reproduction; however, this information was not available in the GO database for ASM223467v1. Similarly, one gene related to “behavior”, Pappalysin 1 (*pappaa*), were identified on chromosomes 12 (Table S6).

## 4 Discussion

### 4.1 Fecundity Increases at Higher Latitudes

Species distributed at high latitudes tend to have higher fecundity, even if they are of the same size (Pincheira‐Donoso and Hunt 2017; Sparks et al. 2022, Álvarez‐Noriega et al. 2023; Figure 2). This tendency is not limited to fishes (Winemiller and Rose 1992; Vila‐Gispert, Moreno‐Amich, and Garcia‐Berthou 2002; Álvarez‐Noriega et al. 2023), and is found in a variety of taxa (Pincheira‐Donoso and Hunt 2017, Tarr et al. 2019, Sparks et al. 2022). In addition, numerous high‐latitude fish species have been reported to increase the number of eggs that they produce in a manner that is disproportionate to body size (Álvarez‐Noriega et al. 2023). Geographic variation in fecundity is considered to reflect an adaptive advantage to higher latitude environments, for example, higher mortality and/or larger temporal change of food availability in more seasonal environments (i.e., higher latitudes) (Pincheira‐Donoso and Hunt 2017; Tarr et al. 2019; Álvarez‐Noriega et al. 2023). However, unlike studies on fruit flies, numerous studies on wild animals encounter challenges in distinguishing between the relative contributions of genetic and environmental variations (Bunger et al. 2005; Green and Extavour 2014). Our study; however, demonstrates the existence of interspecific variation in the size‐fecundity relationship and suggests a potential genetic basis in medaka (Figures 2 and 3). Specifically, *O. sakaizumii* females from Aomori exhibit genetically higher egg production compared to their congener, *O. latipes* from Okinawa (Figures 2b and 3). This system provides a valuable foundation for clarifying the genetic and physiological mechanisms underlying the adaptive evolution of life‐history traits.

The Aomori population exhibits a markedly shorter spawning season compared to the Okinawa population in their natural habitat (Figure 2c,d). At higher latitudes, where the period suitable for reproduction tends to be short, natural selection favors life‐history strategies that maximize the number of offspring per reproductive event (Conover and Schultz 1995; Kokita 2004; Pincheira‐Donoso and Hunt 2017; Tarr et al. 2019). *O. sakaizumii* occupies one of the highest latitudinal ranges in the genus *Oryzias* (e.g., Sumarto et al. 2020). Consistent with the paradigm of adaptive evolution at higher latitudes, *O. sakaizumii* may have undergone evolutionary changes towards increased fecundity as this species expanded its northernmost range (Takehana et al. 2003; Katsumura et al. 2019).

The concepts of thermal plasticity in gonad maturity provides support for the adaptive evolution of reproductive physiology in medaka fishes. Following sexual maturation in females, lower water temperature plastically suppresses gonad maturation and reduces egg production in both *O. latipes* (Hirshfield 1980; Koger et al. 1999; Hemmer‐Brepson et al. 2014) and *O. sakaizumii* (Shinomiya et al. 2023), as demonstrated in temperature‐controlled experiments. In addition, habitat temperatures are typically lower at high latitudes, for example, the average water temperature in Aomori during the spawning season is 15.7°C ± 4.3°C (from May 1, 2013 to July 26, 2013), and that of Okinawa is 22.3°C ± 3.4°C (from March 8, 2012 to October 15, 2012) (see also Yamahira et al. 2007; Shinomiya et al. 2023). Consequently, the observation that Aomori females produce more eggs than Okinawa females in their natural environment (Figure 2a), contrary to the pattern anticipated from thermal plasticity, suggests adaptive evolution in fecundity. Such counter‐gradient variation in fecundity traits has been reported in latitudinal populations of several fishes and birds (e.g., Conover and Schultz 1995, Kokita 2004; Pincheira‐Donoso and Hunt 2017; Sparks et al. 2022).

### 4.2 Genetic Basis of the Size‐Fecundity Relationship

From an individual perspective, ontogenetic changes or plasticity in reproductive investment represent critical factors in enhancing an individual's lifetime reproductive success (Kooijman 2010; Simmons, Lupold, and Fitzpatrick 2017; White et al. 2022). The regulation of egg production through nutrient metabolism may constitute one of the physiological mechanisms underlying ontogenetic changes in reproductive investment (Copeland et al. 2011; Dantzer and Swanson 2012). For example, in *Drosophila* species, *igf1* expression plastically alters ovariole number in response to food availability (Green and Extavour 2014). Furthermore, a GWAS study conducted in chickens showed that *igf1* exhibits pleiotropic effects on both body weight and egg production (Wolc et al. 2014). Interestingly, a transgenic medaka strain that overexpress growth hormone and *igf1* shows faster somatic growth, but lower fecundity compared to the wild type (Komine et al. 2016). In our study, the QTL for egg number on chromosome 23 includes both *igf1* and *lep‐b* (Table 1; Table S4). Both IGF signaling genes and leptin are associated with somatic growth and gonad development in vertebrates (Copeland et al. 2011; Dantzer and Swanson 2012; Chisada et al. 2014; Ndandala et al. 2022), suggesting that these genes may influence interspecific variations in the size‐fecundity relationship.

In this study, our QTL analysis focused on evaluating egg numbers in mating trials. If spawning fails to occur due to factors such as sexual motivation or mate preference in either of the sexes (e.g., Yokoi et al. 2020; Daimon et al. 2022; Kondo et al. 2023), then the detection of QTLs for egg number may potentially be biased by sexual motivation. However, the QTLs for egg number do not overlap with the QTL peaks associated with female spawning latency or male courtship frequency (Table 1; Table S4; Figure 3). These findings suggest that the observed QTLs for egg numbers are unlikely statistical confounders related to the sexual motivation of F_2_ individuals.

In addition, the peaks of the six QTLs were not consistent between the AFOM and OFAM families (Table S4; Figure S3), which may be due to genetic variation within the population. Since wild individuals were used as grandparents in the QTL analysis, individual differences in genetic background may exist between the two families. All four of the QTLs of associated with mating behaviors tended to show effects opposite to those expected from phenotypic differences in wild populations reported in previous studies (Figure S4, e.g., Kagawa 2014, Fujimoto, Miyake, and Yamahira 2015), further suggesting genetic variation within wild populations. Moreover, genetic studies in medaka reported that sex‐specific alleles, possibly related to sex determination, are distributed across multiple chromosomes (Shinomiya et al. 2010; Kitano et al. 2023; Shinya, Kimura, and Naruse 2023), indicating that males and females of the same population might carry different alleles at these loci. Given the genetic variation within wild populations, further validation is necessary to more comprehensively determine whether the QTLs detected in this study affect phenotypes in a sex‐ and species‐specific manner. This will require enhanced research methods, such as breeding designs with replicates or genome‐wide association studies using improved genetic markers in the wild populations.

In this study, the PVE for egg number calculated with genome‐wide markers was 16.2% and 28.2% for OFAM and AFOM, respectively (Table S4). The PVE values were similar to the heritability of 0.24 obtained in a genome‐wide association study of salmonids using 57,000 SNPs (D'Ambrosio et al. 2020). QTL and GWAS studies conducted in domesticated animals have revealed that size and fecundity are often regulated by distinct chromosomal regions (Bunger et al. 2005; Minvielle et al. 2005; Wright et al. 2010; Wolc et al. 2014; D'Ambrosio et al. 2020). Similarly, in medaka, numerous QTLs associated with body size do not overlap with QTLs for egg number. For instance, the QTL for body weight is located on chromosome 4 (Yassumoto et al. 2020), and the QTLs for SL are located on chromosomes 12 and 24 (Table 1; Kawajiri et al. 2014). Such a genetic architecture, characterized by multiple genetically independent chromosomal regions, may facilitate the adaptive evolution of the size‐fecundity relationship.

### 4.3 Relationship Between Fecundity Evolution and Proportion of Spawning Females

We observed differences in the proportion of spawning females among wild populations (Figure 2c,d; Figure S2). This difference in spawning among females in the wild is primarily attributed to the higher frequency of spawning in the Aomori females compared to the Okinawa females (Figure S2), resulting in a less male‐biased operational sex ratio (OSR) in the Aomori population (i.e., the number of spawned females to the total number of adult males and females). Since the numbers of adult males and females being nearly equal in both populations (Table S1), the proportion of spawning females significantly influences the bias in OSR. On average, the Aomori population tends to exhibit a less male‐biased OSR (0.65 ± 0.13; Figure 2c) compared to the Okinawa population (0.75 ± 0.12; Figure 2d) throughout the spawning season. These results suggest that fecundity evolution affects OSR as a by‐product of life‐history adaptation.

Few reports have investigated whether OSR varies with environmental gradients, such as latitude and/or altitude (Hettyey et al. 2005; Monteiro and Lyons 2012; Petry et al. 2016; Machado et al. 2016; Monteiro et al. 2017; Garcia‐Roa et al. 2020). In a case study on pipefish, sex‐specific thermal plasticity in reproduction has been proposed as a physiological mechanism driving variations in OSR among latitudinal populations (Monteiro and Lyons 2012; Monteiro et al. 2017). Although our findings initially appear to align with those of the pipefish study, thermal plasticity in medaka does not account for the observed spawning intervals at higher latitudes with cooler climates (Hirshfield 1980; Koger et al. 1999; Hemmer‐Brepson et al. 2014). Consequently, the disparities in OSR among medaka populations should be attributed to the evolutionary changes in female reproductive traits, such as spawning interval (Figure S1) (e.g., Montenegro et al. 2022) and/or the synchronous reproduction of *O. sakaizumii* females in specific seasons (Figure 2c,d) (Shinomiya et al. 2023). Evolutionary shifts in female reproductive traits along environmental gradients have been extensively reported in several animal species (Pincheira‐Donoso and Hunt 2017; Álvarez‐Noriega et al. 2023). Given that females tend to be more sensitive to environmental cues than males in terms of reproductive decisions (Morbey and Ydenberg 2001; Nakazawa and Hsu 2023), adaptations in female reproductive life‐history may significantly influence OSR along environmental gradients in other animal taxa as well.

OSR typically affects the variance that exists in reproductive success, thereby influencing opportunities for sexual selection through individual sexual interactions (Shuster and Wade 2003; Klug et al. 2010; Janicke and Morrow 2018), including mating competition and mate choice (e.g., Grant, Bryant, and Soos 1995; Grant, Gaboury, and Levit 2000; Forsgren et al. 2004; Sogabe and Yanagisawa 2007). In some animal species, including medaka (Fujimoto, Miyake, and Yamahira 2015; Sumarto et al. 2021); sexual selection pressure is hypothesized to be weaker at higher latitudes, as indicated by latitudinal variations in sexual dimorphism (Fujimoto, Miyake, and Yamahira 2015; Tarr et al. 2019; Sumarto et al. 2020; Dudaniec et al. 2022; Murray et al. 2021) and mating behavior (Fujimoto, Miyake, and Yamahira 2015; Sumarto et al. 2021; Matsumura et al. 2023). Our findings underscore that the reduced male bias in OSR observed at higher latitudes serves as one of the ecological mechanisms contributing to weaker sexual selection at higher latitudes in ricefish.

## 5 Conclusion

We demonstrated interspecific variation in the size‐fecundity relationship in two medaka species. Since *O. sakaizumii* is distributed at higher latitudes and has a shorter spawning season than *O. latipes* in the wild, we propose that adaptation to high latitudes is responsible for the relatively high fecundity observed in *O. sakaizumii*. QTL analysis using interspecific F_2_ hybrids showed that chromosome 23 is linked to the size‐fecundity relationship, supporting the genetic basis and fecundity evolution in high‐latitude females. Interestingly, fecundity evolution also results in a less male‐biased OSR as a by‐product of life‐history adaptation in seasonal reproduction. These findings suggest that life‐history evolution along latitudinal gradients significantly affects the reproductive characteristics of wild medaka populations.

## Author Contributions

S.F., M.Y., and K.Y. conceived and designed the study. S.F., S.T., and K.Y. performed field investigations. S.F., T.M., D.M.F., I.M., B.K.A.S., and K.Y. performed laboratory analyses. S.F., T.M., S.A., and J.K. conducted the analyses. S.F. and K.Y. wrote the first draft. S.F. wrote the submitted manuscript. All authors read and approved the final manuscript.

## Ethics Statement

All experimental methods related to the handling of live fish were carried out in accordance with the Regulations for Animal Experiments at the University of the Ryukyus. Experiments were approved by the Animal Care Ethics Committee of University of the Ryukyus (Approval No. A2021012). The collection and rearing experiments involving wild *Oryzias latipes* in Okinawa Prefecture was conducted in accordance with the Protection Ordinance of Precious wild animals and plants in Okinawa Prefecture (No. 92, 2021/4/26).

## Conflicts of Interest

The authors declare no conflicts of interest.

## Supporting information


Data S1.

## Data Availability

Analyzed data and custom R scripts have been deposited in Dryad. https://doi.org/10.5061/dryad.mpg4f4r7d.
