# Supplementary material for: Evolution of Size‐Fecundity Relationship in Medaka Fish From Different Latitudes
Source: Mol Ecol. 2024 Nov 5;33(23):e17578. doi: 10.1111/mec.17578 (PMC11589666; doi:10.1111/mec.17578)
Supplement: Supplementary file 1 — Data S1. [file MEC-33-e17578-s001.doc]

**Evolution of size-fecundity relationship in medaka fish from different latitudes**

Shingo Fujimoto^1,2,3^, Bayu K. A. Sumarto^3,4^, Iki Murase^3^, Daniel F. Mokodongan^3,5^, Taijun Myosho^6^, Mitsuharu Yagi^7^, Satoshi Ansai^8,9,10^, Jun Kitano^8^, Satoshi Takeda^11^, Kazunori Yamahira^3^

^1^ Integrated Technology Center, University of the Ryukyus, Okinawa 903-0125, Japan. (Present address)

^2^ Research Laboratory Center, Faculty of Medicine, University of the Ryukyus, Okinawa 903-0213, Japan.

^3^ Tropical Biosphere Research Center, University of the Ryukyus, Okinawa 903-0213, Japan.

^4^ Research Center for Conservation of Marine and Inland Water Resources, National Research and Innovation Agency, 16915, Indonesia (Present address)

^5^ Museum Zoologicum Bogoriense (MZB), Research Center for Biosystematics and Evolution, National Research and Innovation Agency (BRIN), Cibinong, Indonesia. (Present address)

^6^ Laboratory of Molecular Reproductive Biology, Institute for Environmental Sciences, University of Shizuoka, Shizuoka 422-8526, Japan.

^7^ Graduate School of Fisheries and Environmental Sciences, Nagasaki University, Nagasaki 852-8521, Japan.

^8^ Ecological Genetics Laboratory, Department of Genomics and Evolutionary Biology, National Institute of Genetics, Mishima, Shizuoka 411-8540, Japan.

^9^ Graduate School of Agriculture, Kyoto University, Kyoto 606-8502, Japan.

^10^ Ushimado Marine Institute, Okayama University, Setouchi, Okayama 701-4303, Japan. (Present address)

^11^ Research Center for Marine Biology, Graduate School of Life Sciences, Tohoku University, Aomori 039-3501, Japan.

**Corresponding author contact details**

Shingo Fujimoto, Integrated Technology Center, University of the Ryukyus, Okinawa 903-0125, Japan, Tel: +81-98-895-1205

E-mail: fujimoto.s@outlook.com

**Supporting information**

Fig. S1. Experimental procedures for spawning assessment in wild populations, F2 crosses, and stock individuals for QTL analysis

Fig. S2. Comparison of the number of egg clutches produced by females during 2-day mating trials in the spawning season in Okinawa and Aomori. Colors represent the following categories: two clutches (pale gray), a single clutch (dark gray), no clutch (black).

Fig. S3. QTL analysis of the F_2_ hybrids between Okinawa and Aomori. (a) Logarithm of the odd (LOD) score for the phenotypes in F_2_ females derived from F_1_ hybrids between an Aomori female and an Okinawa male (AFOM, red lines), (b) LOD score for the phenotypes in AFOM F_2_ males, (c) LOD score for the phenotypes in F_2_ females derived from F_1_ hybrids between an Okinawa female and an Aomori male (OFAM, blue lines), (d) LOD score for the phenotypes in OFAM F_2_ males. Lines indicate significance levels of the false discovery rate (FDR) from genome-wide permutation tests with multiple peaks (solid line: FDR < 0.05, dotted line: 0.05 < FDR < 0.10).

Fig. S4. Effect plot of six QTL loci on each phenotype (OK: Okinawa allele, AO: Aomori allele). (a) Standard length of genotypes of the single nucleotide polymorphism, marker OL_C12_17565800. (b) courtship frequency of approaching, OL_C20_20494125. (c) wrapping rejection, OL_C24_8163224 (d) average egg number, OL_C23_190103934. (e) spawning latency, OL_C13_12323836, and (f) spawning latency, OL_C18_4309373.

Table S1. Comparison of the total number of individuals, males, females, juvenile individuals, adult sex ratios (ASR; ratio of males to total adults), and *P* values in Okinawa and Aomori (binomial test for deviation of ASR from 0.5, significance levels: Okinawa, *P* < 0.0046; Aomori, *P* < 0.01).

Table S2. Number of individuals used in the mating trials for the spawning season assessment.

Table S3. Comparison of generalized additive model results to estimate seasonal trends in the proportion of mature males and females. AIC: Akaike’s information criterion.

Table S4. List of all phenotypes examined for QTL analysis.

Table S5. Top 10 gene ontology terms obtained from GO enrichment analysis, sorted by ascending enrichment FDR.

Table S6. Candidate genes involved in behavior, oogenesis, and reproduction.


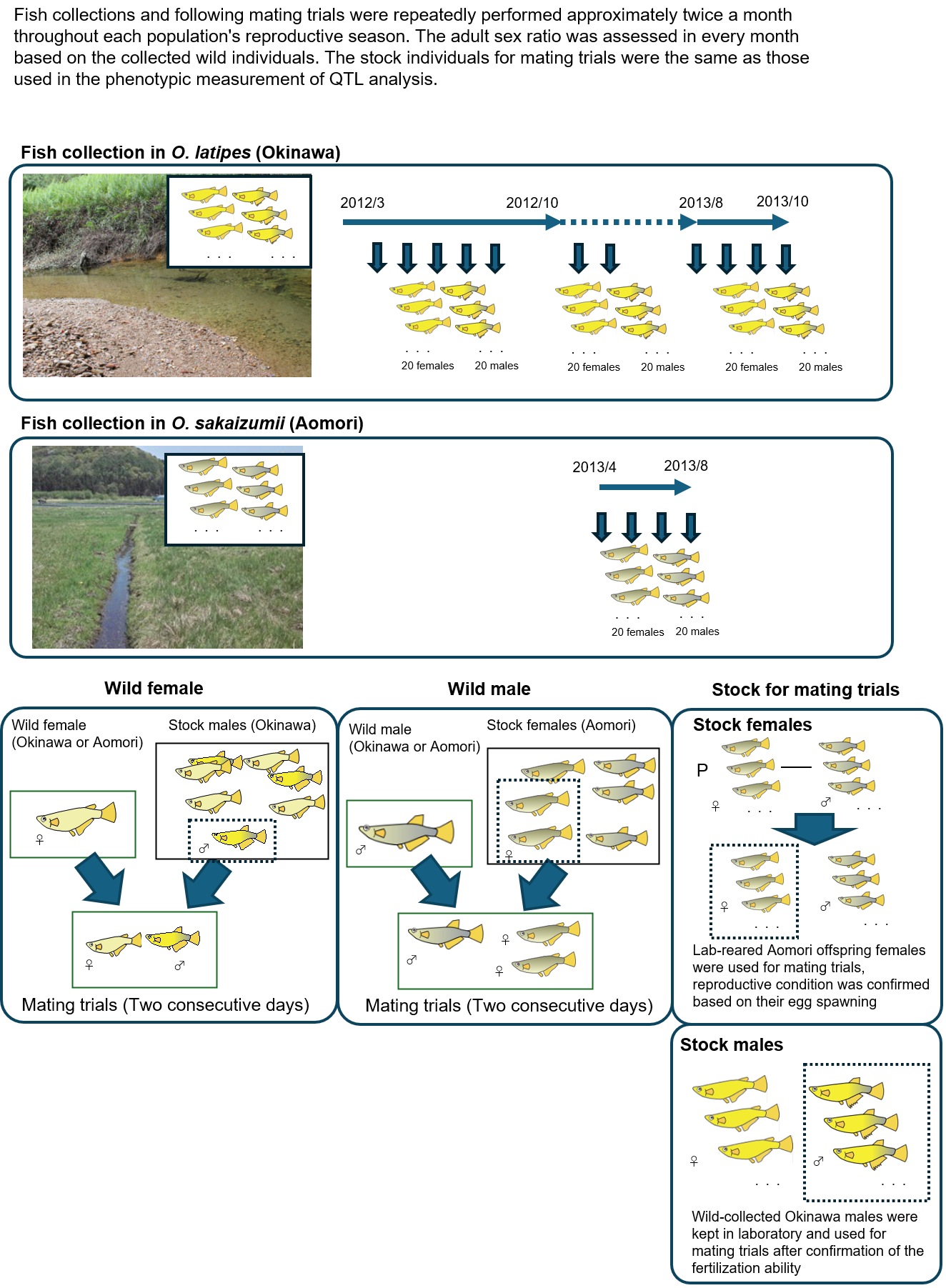


Fig. S1. Experimental procedures for spawning assessment in wild populations, F2 crosses, and stock individuals for QTL analysis


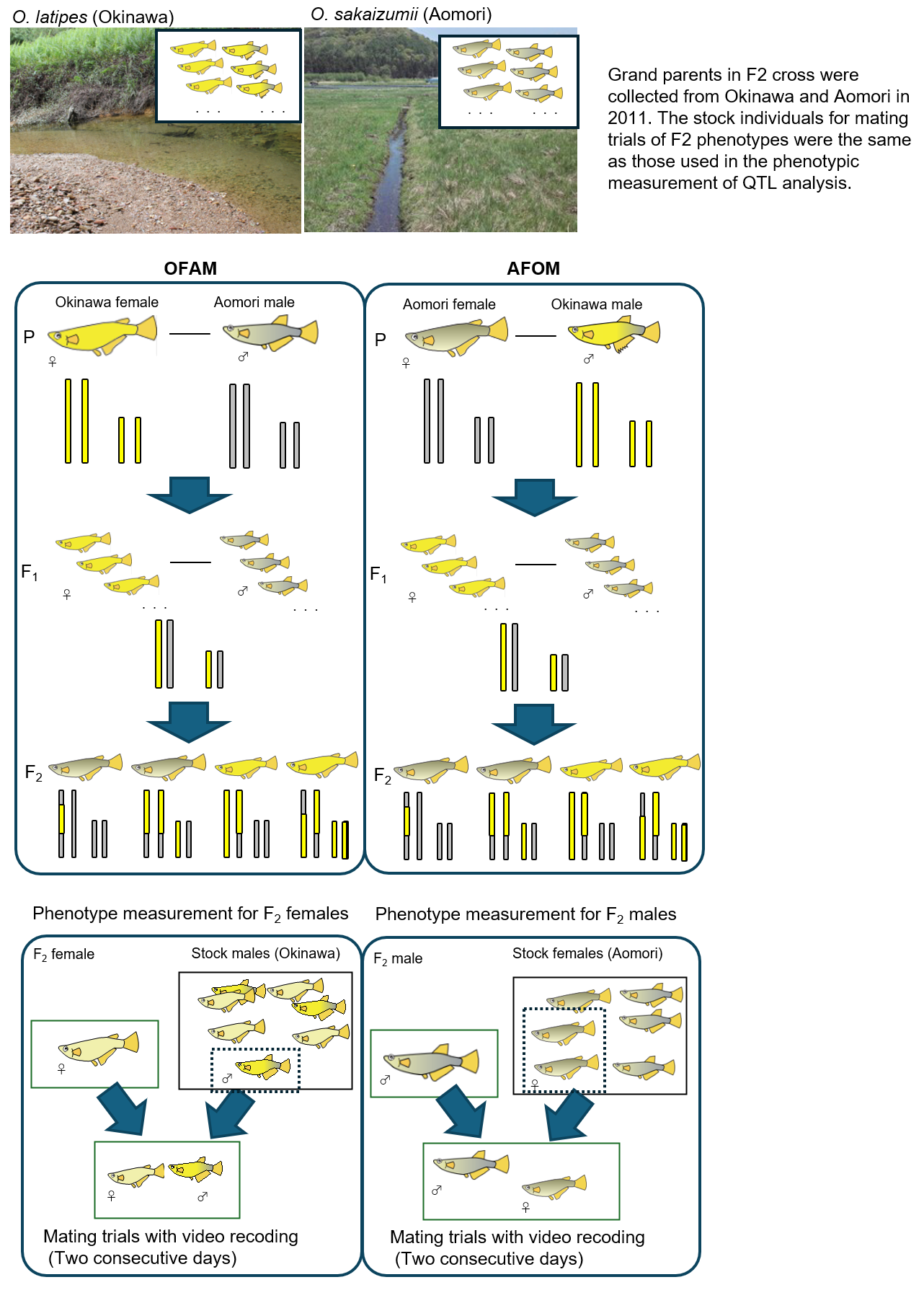


Fig. S1. (Continued)


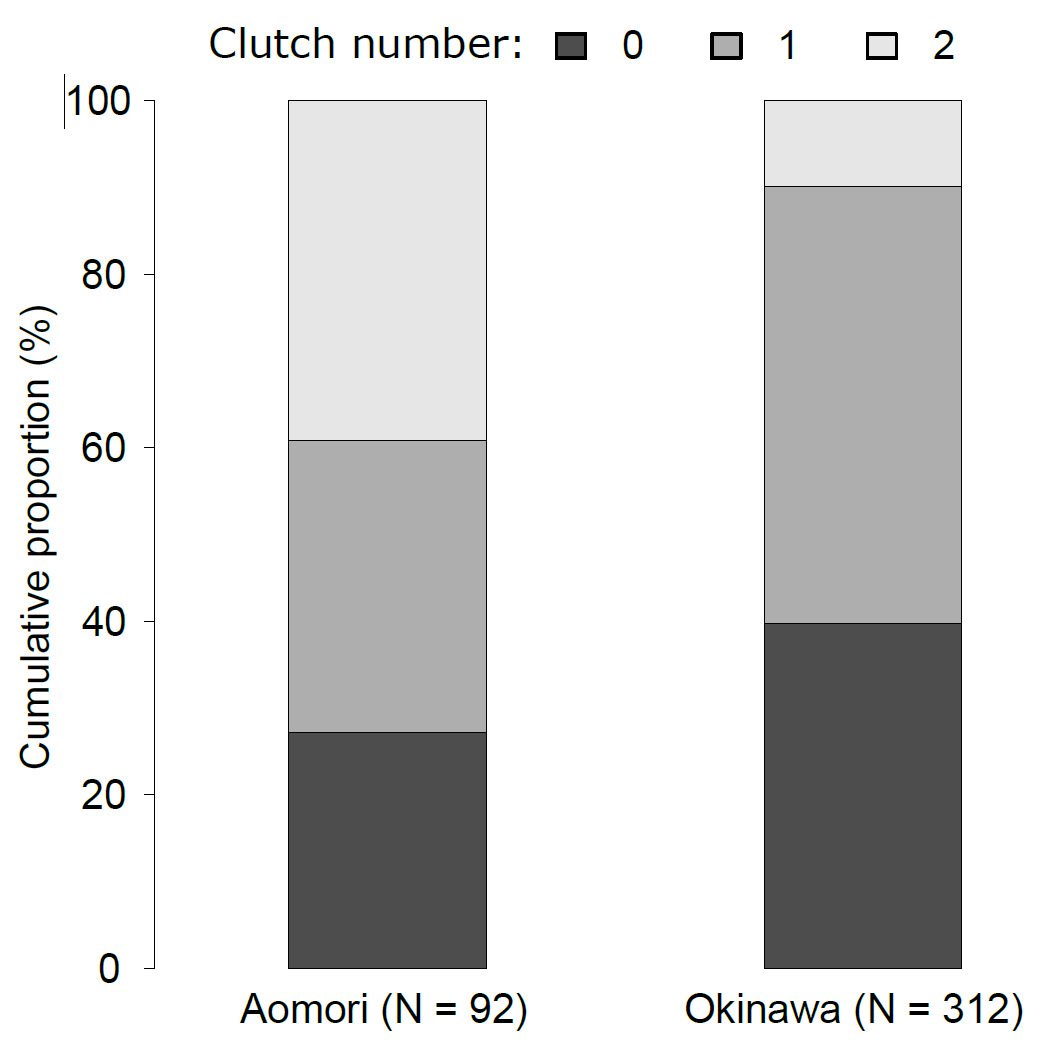


Fig. S2. Comparison of the number of egg clutches produced by females during 2-day mating trials in the spawning season in Okinawa and Aomori. Colors represent the following categories: two clutches (pale gray), a single clutch (dark gray), no clutch (black).


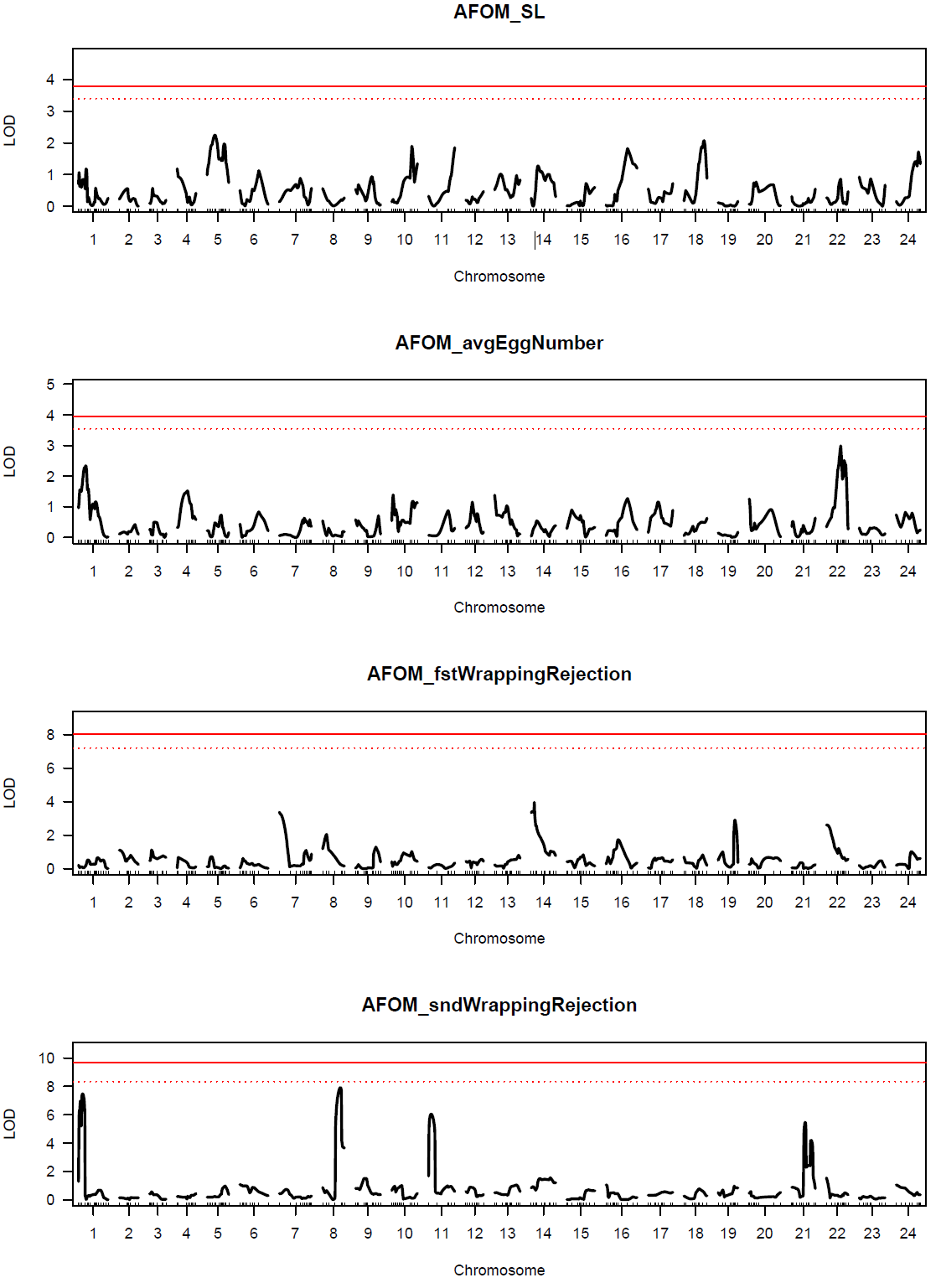


Standard length (AFOM female)

a

Average egg number (AFOM female)

Wrapping rejection in 1st trial (AFOM female)

Wrapping rejection in 2nd trial (AFOM female)

Fig. S3. QTL analysis of the F_2_ hybrids between Okinawa and Aomori. (a) Logarithm of the odd (LOD) score for the phenotypes in F_2_ females derived from F_1_ hybrids between an Aomori female and an Okinawa male (AFOM, red lines), (b) LOD score for the phenotypes in AFOM F_2_ males, (c) LOD score for the phenotypes in F_2_ females derived from F_1_ hybrids between an Okinawa female and an Aomori male (OFAM, blue lines), (d) LOD score for the phenotypes in OFAM F_2_ males. Lines indicate significance levels of the false discovery rate (FDR) from genome-wide permutation tests with multiple peaks (solid line: FDR < 0.05, dotted line: 0.05 < FDR < 0.10).

a


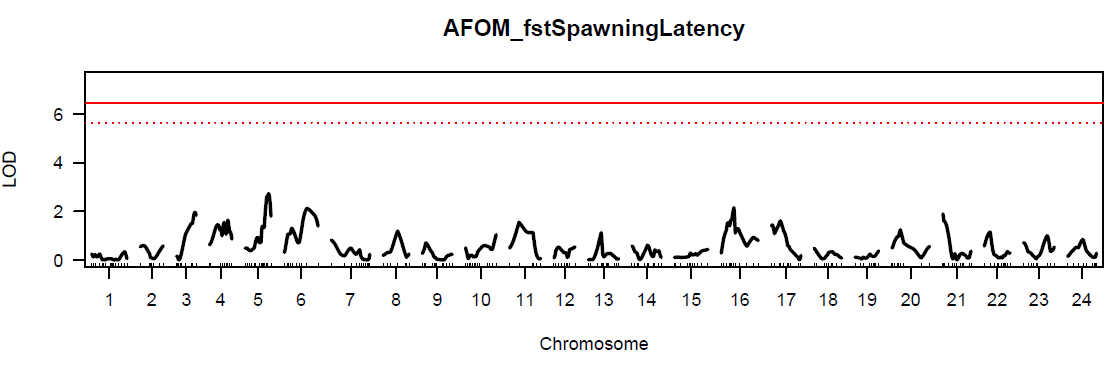


Spawning latency in 1st trial (AFOM female)

Spawning latency in 2nd trial (AFOM female)


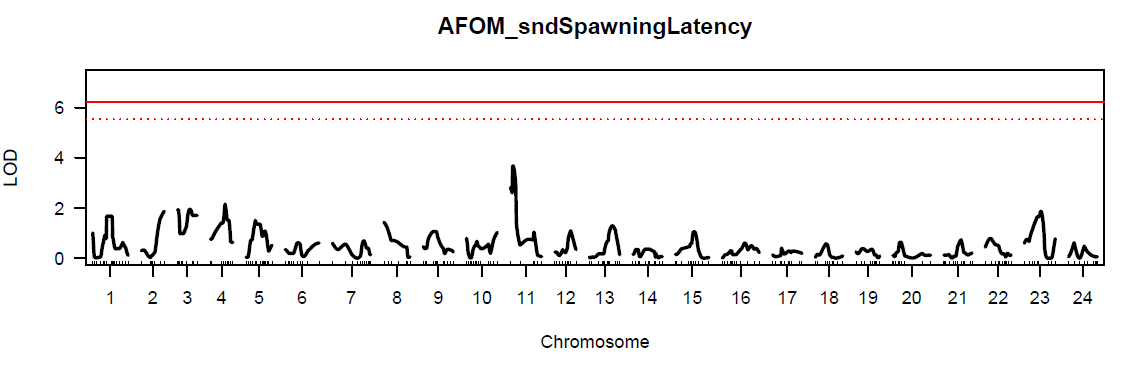


Fig. S3. (Continued)


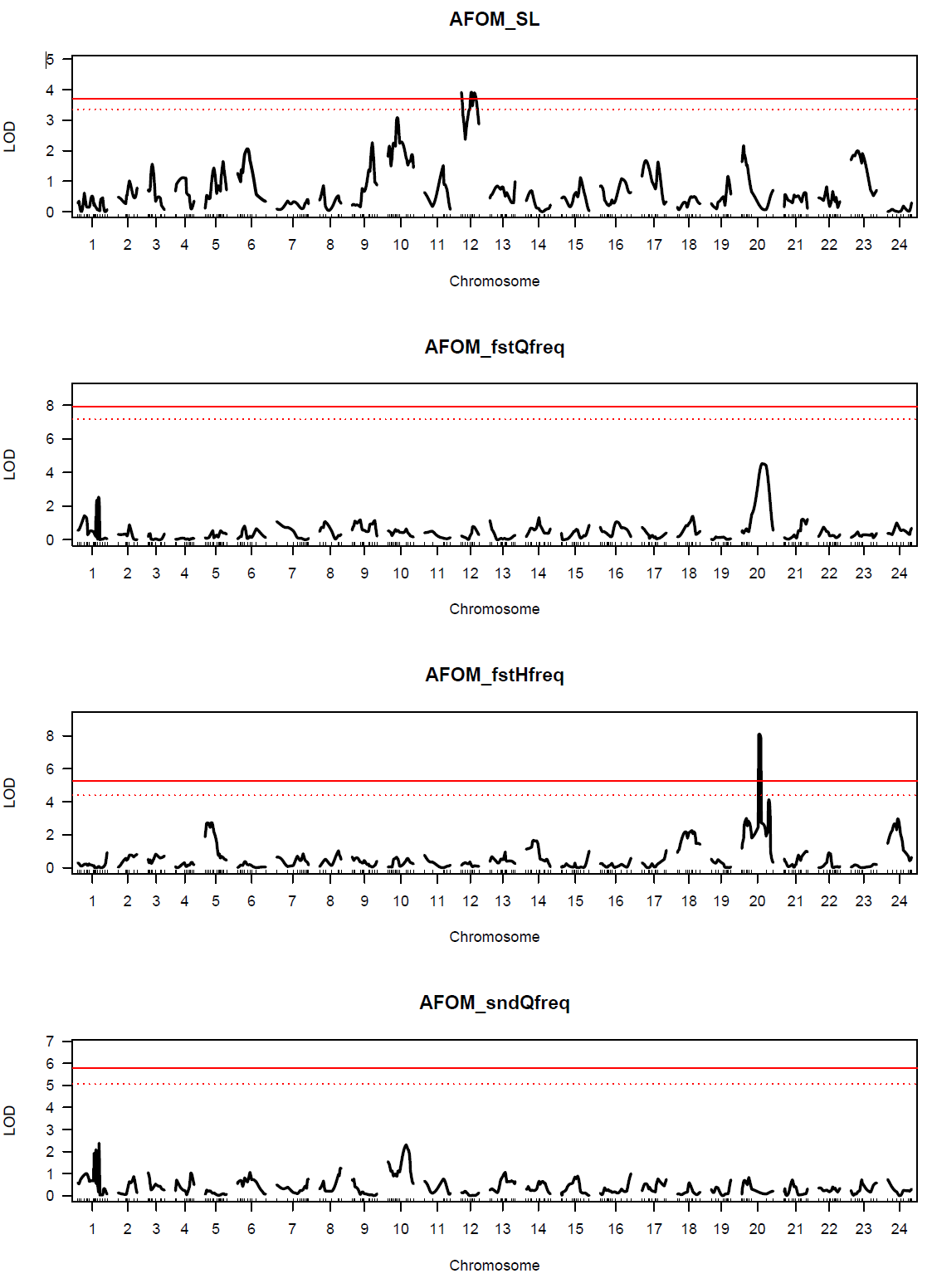


Courtship frequency of quick circle in 1st trial (AFOM male)

b

Standard length (AFOM male)

Courtship frequency of approaching in 1st trial (AFOM male)

Courtship frequency of quick circle in 2nd trial (AFOM male)

Courtship frequency of approaching in 2nd trial (AFOM male)


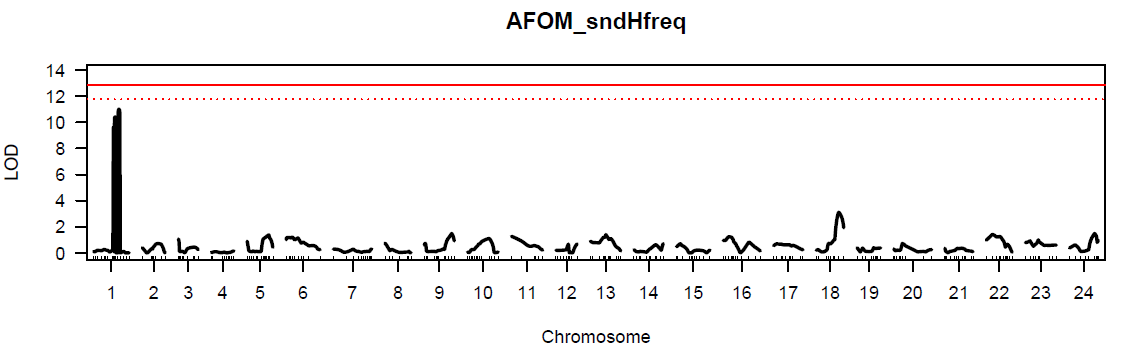


Fig. S3. (Continued)


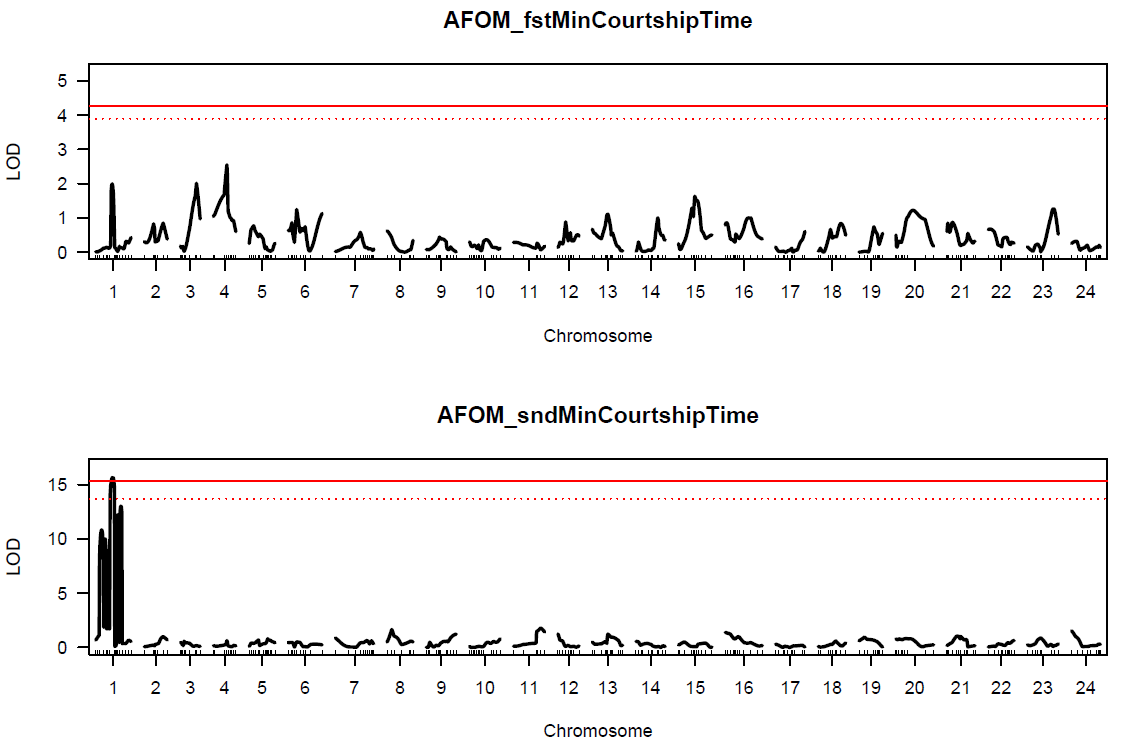


Courtship latency in 1st trial (AFOM male)

Courtship latency in 2nd trial (AFOM male)

b

Fig. S3. (Continued).


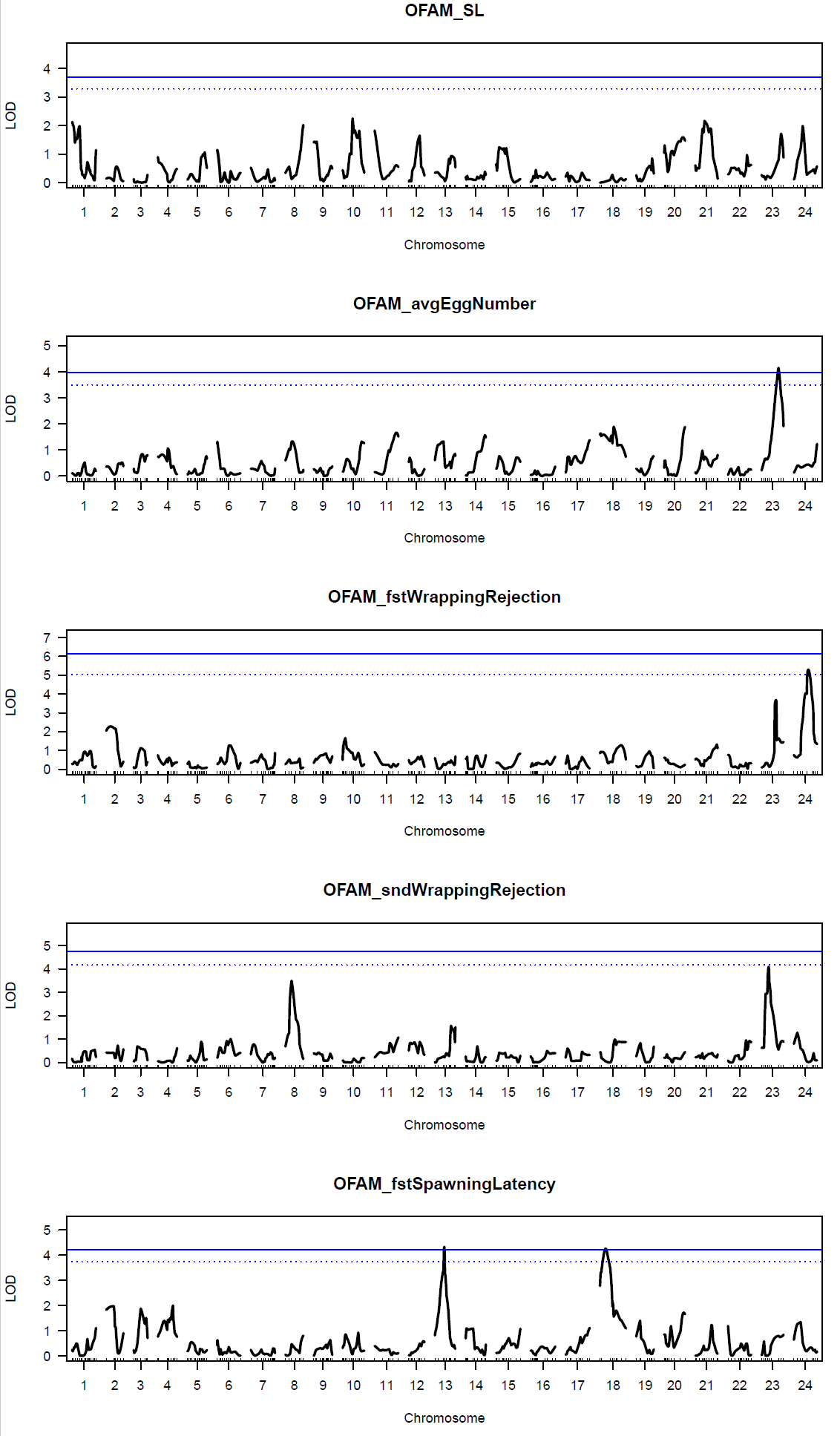

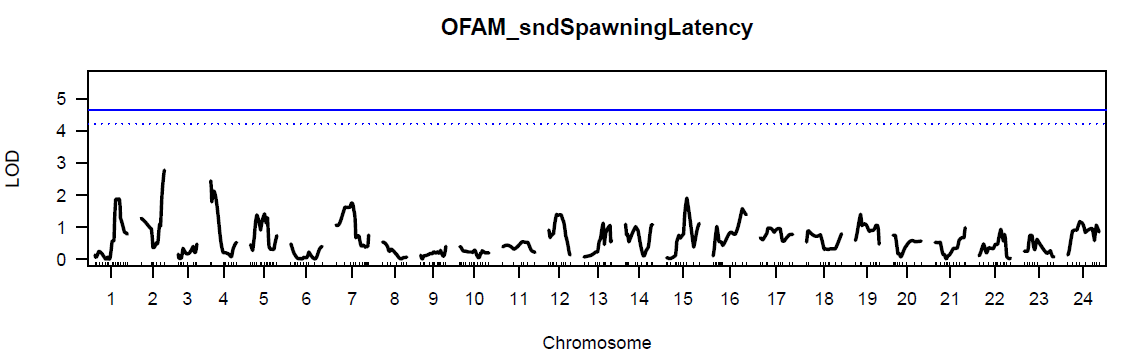


c

Standard length (OFAM female)

Average egg number (OFAM female)

Wrapping rejection in 1st trial (OFAM female)

Wrapping rejection in 2nd trial (OFAM female)

Spawning latency in 1st trial (OFAM female)

Spawning latency in 2nd trial (OFAM female)

Fig. S3. (Continued).

d

Standard length (OFAM male)


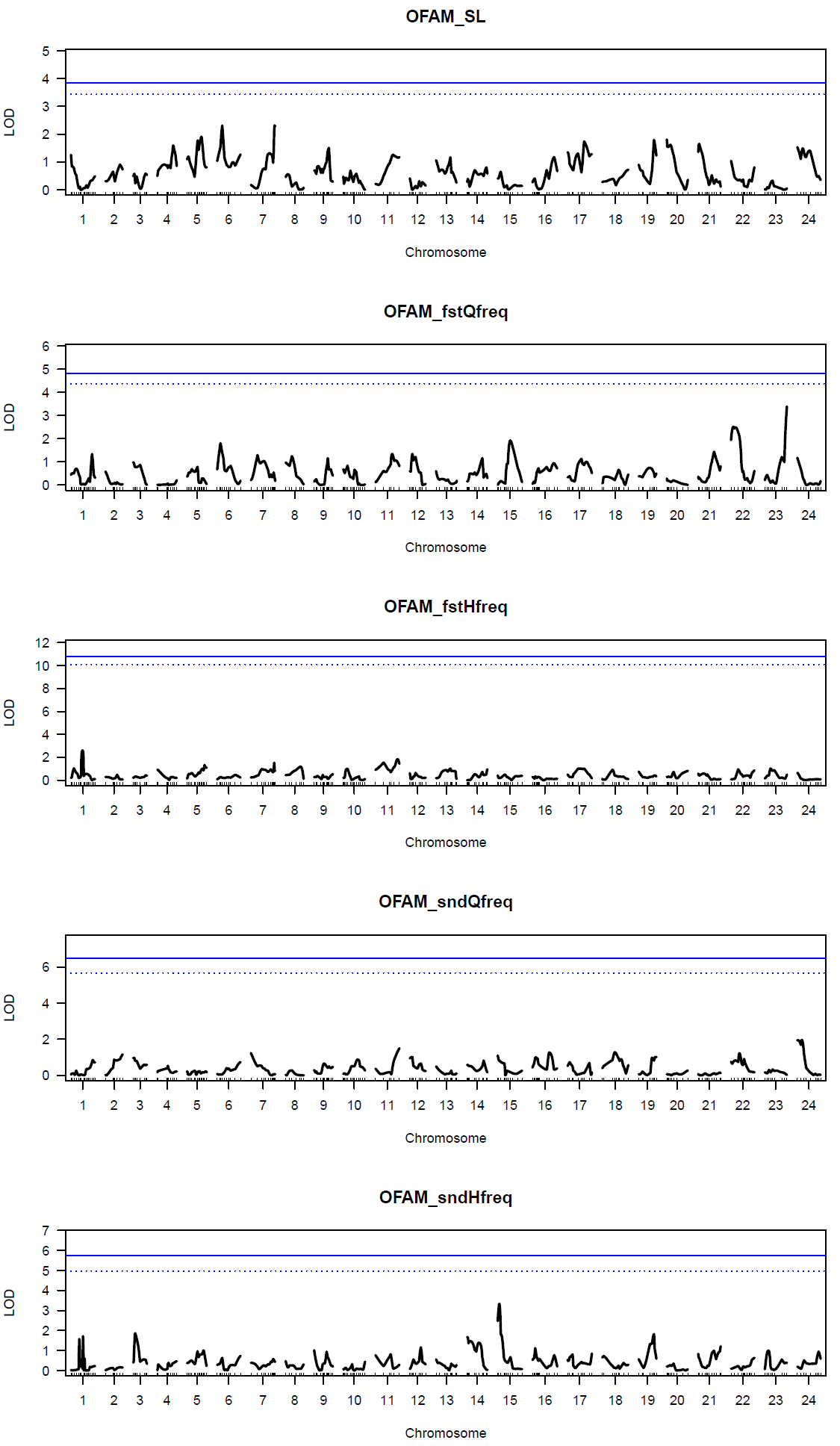


Courtship frequency of quick circle in 1st trial (OFAM male)

Courtship frequency of approaching in 1st trial (OFAM male)

Courtship frequency of quick circle in 2nd trial (OFAM male)

Courtship frequency of approaching in 2nd trial (OFAM male)

Fig. S3. (Continued).


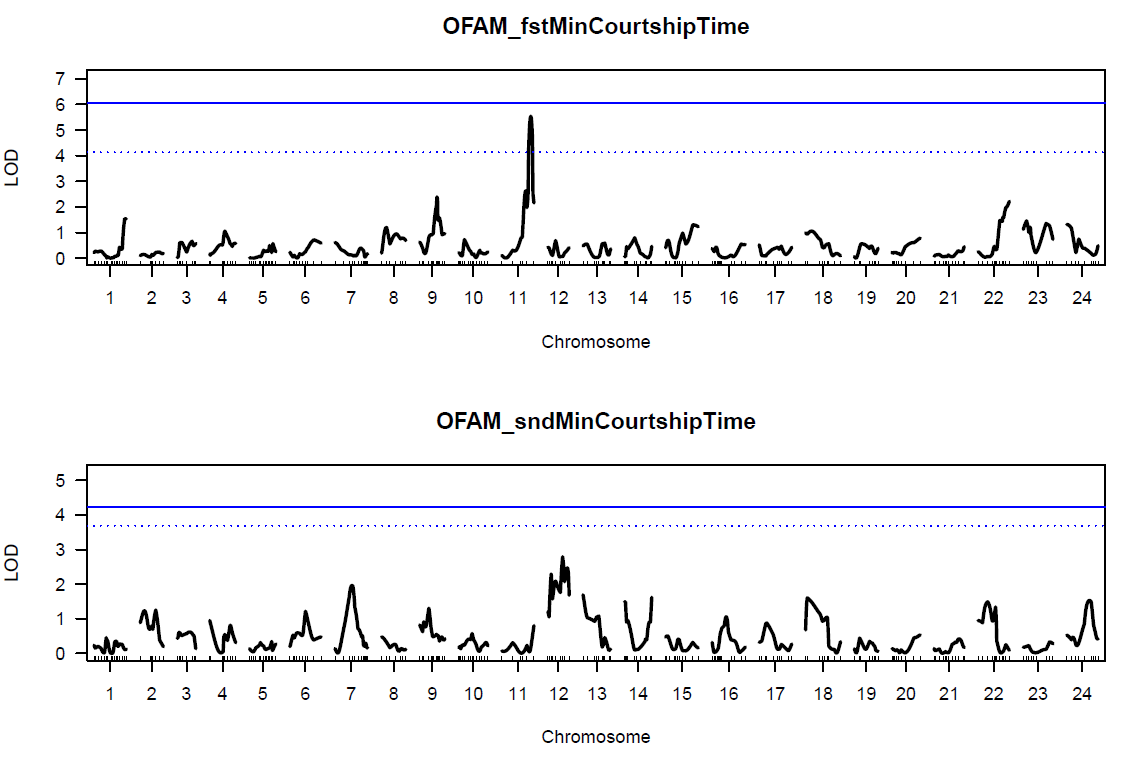


Courtship latency in 1st trial (OFAM male)

Courtship latency in 2nd trial (OFAM male)

d


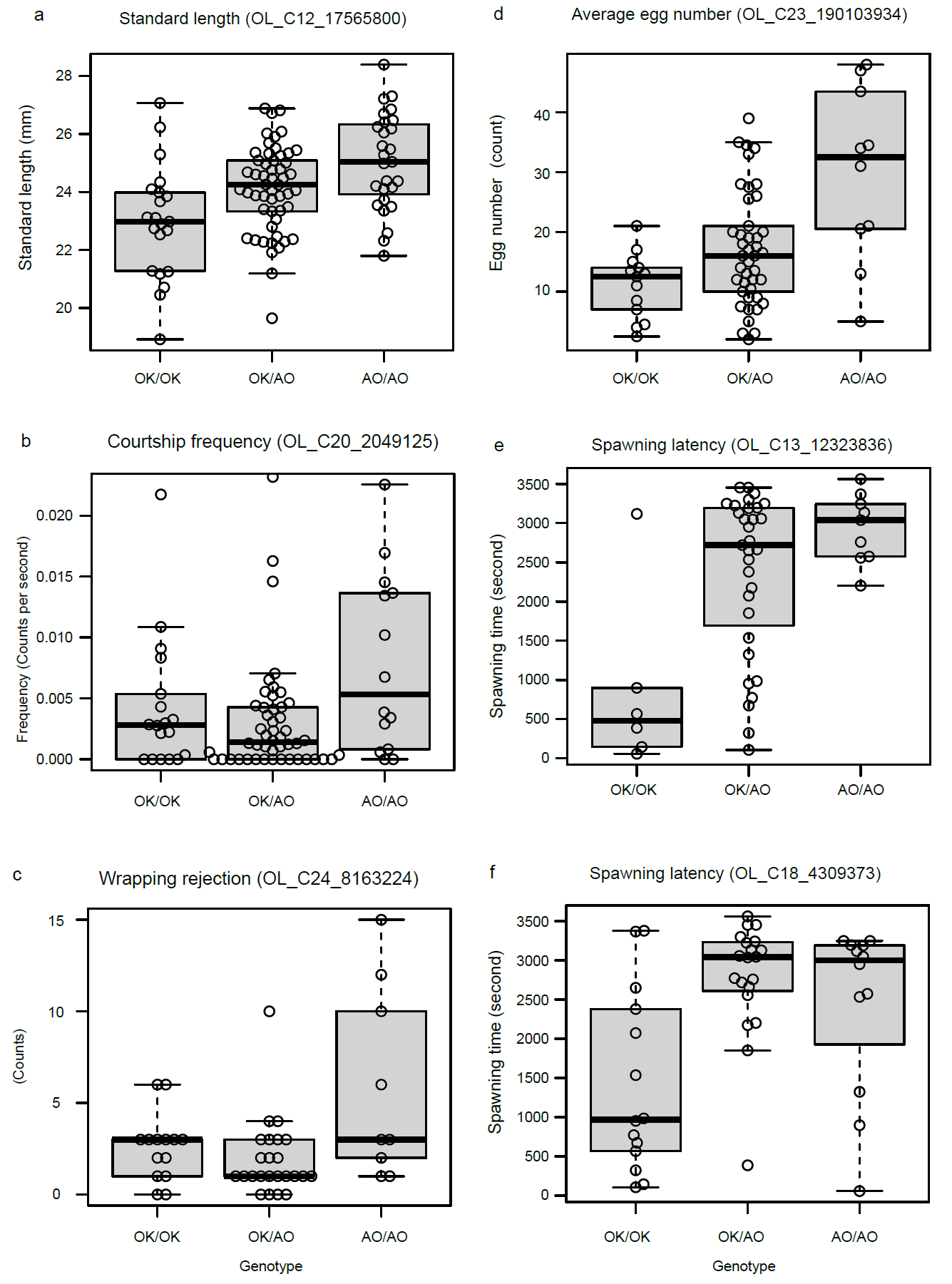


Fig. S4. Effect plot of six QTL loci on each phenotype (OK: Okinawa allele, AO: Aomori allele). (a) Standard length of genotypes of the single nucleotide polymorphism, marker OL_C12_17565800. (b) courtship frequency of approaching, OL_C20_20494125. (c) wrapping rejection, OL_C24_8163224 (d) average egg number, OL_C23_190103934. (e) spawning latency, OL_C13_12323836, and (f) spawning latency, OL_C18_4309373.

Table S1. Comparison of the total number of individuals, males, females, juvenile individuals, adult sex ratios (ASR; ratio of males to total adults), and *P* values in Okinawa and Aomori (binomial test for deviation of ASR from 0.5, significance levels: Okinawa, *P* < 0.0046; Aomori, *P* < 0.01).

| Population | Date | Total | Male | Female | Juvenile | ASR | *P* value |
| --- | --- | --- | --- | --- | --- | --- | --- |
| Okinawa | 2012/03/24 | 269 | 127 | 97 | 45 | 0.57 | 0.052 |
|  | 2012/04/18 | 250 | 100 | 92 | 58 | 0.52 | 0.613 |
|  | 2012/05/12 | 164 | 90 | 70 | 4 | 0.56 | 0.133 |
|  | 2012/06/11 | 198 | 87 | 70 | 41 | 0.55 | 0.201 |
|  | 2012/07/12 | 446 | 44 | 40 | 362 | 0.52 | 0.743 |
|  | 2012/08/12 | 399 | 124 | 99 | 176 | 0.56 | 0.108 |
|  | 2012/09/14 | 743 | 162 | 151 | 430 | 0.52 | 0.572 |
|  | 2012/10/25 | 687 | 180 | 197 | 310 | 0.48 | 0.410 |
|  | 2012/12/04 | 1472 | 558 | 566 | 348 | 0.50 | 0.835 |
|  | 2013/02/13 | 484 | 163 | 216 | 105 | 0.43 | 0.007 |
|  | 2013/03/18 | 446 | 178 | 209 | 59 | 0.46 | 0.127 |
| Aomori | 2013/04/29 | 119 | 35 | 33 | 51 | 0.51 | 0.904 |
|  | 2013/05/28 | 129 | 44 | 73 | 12 | 0.38 | 0.009 |
|  | 2013/06/29 | 349 | 32 | 48 | 269 | 0.40 | 0.093 |
|  | 2013/07/26 | 543 | 23 | 33 | 487 | 0.41 | 0.229 |
|  | 2013/08/15 | 655 | 201 | 191 | 263 | 0.51 | 0.650 |

Table S2. Number of individuals used in the mating trials of the spawning season assessment

| Population | Date | Male | | Female | |
| --- | --- | --- | --- | --- | --- |
|  |  | *N* | Reproduction successful | *N* | Reproduction successful |
| Okinawa | 2012/03/07 | 20 | 12 | 19 ^*1^ | 4 |
|  | 2012/03/24 | 20 | 14 | 20 | 5 |
|  | 2012/04/04 | 20 | 17 | 19 ^*1^ | 3 |
|  | 2012/04/18 | 20 | 15 | 19 ^*1^ | 1 |
|  | 2012/05/12 | 20 | 20 | 20 | 18 |
|  | 2012/05/18 | 20 | 15 | 20 | 8 |
|  | 2012/05/29 | 20 | 20 | 20 | 17 |
|  | 2012/06/11 | 20 | 19 | 20 | 14 |
|  | 2012/06/26 | 20 | 20 | 20 | 18 |
|  | 2012/07/12 | 20 | 20 | 20 | 16 |
|  | 2012/07/25 | 20 | 20 | 20 | 20 |
|  | 2012/08/12 | 20 | 20 | 19 ^*2^ | 18 |
|  | 2012/09/11 | 20 | 20 | 20 | 0 |
|  | 2012/09/26 | 20 | 20 | 20 | 0 |
|  | 2012/10/17 | 20 | 17 | 18 ^*1, *2^ | 0 |
|  | 2012/12/27 | 20 | 10 | 20 | 0 |
|  | 2013/02/13 | 18 ^*1, *3^ | 6 | 19 ^*1^ | 0 |
|  | 2013/08/21 | - | - | 20 | 19 |
|  | 2013/08/31 | - | - | 16 ^*1^ | 8 |
|  | 2013/09/14 | - | - | 20 | 15 |
|  | 2013/10/03 | - | - | 20 | 4 |
|  | 2013/10/18 | - | - | 19 ^*3^ | 0 |
| Aomori | 2013/04/15 | 18 ^*3^ | 0 | 19 ^*3^ | 0 |
|  | 2013/04/29 | 20 | 0 | 19 | 0 |
|  | 2013/05/13 | 20 | 14 | 20 | 4 |
|  | 2013/05/28 | 20 | 20 | 17^*1^ | 16 |
|  | 2013/06/10 | 20 | 20 | 20 | 20 |
|  | 2013/06/29 | 20 | 20 | 20 | 14 |
|  | 2013/07/14 | 20 | 20 | 15^*1^ | 13 |
|  | 2013/08/03 | 20 | 8 | 18 | 0 |

Asterisks indicate why individuals were excluded from the statistical analyses (^*1^; Experimental fish died during 2-day observation period, ^*2^; Misidentification of sex, ^*3^; individuals <20 mm standard length)

Table S3. Comparison of generalized additive model results to estimate seasonal trends in the proportion of mature males and females.

| Model name | Parameters | Residual degrees of freedom | Deviance explained (%) | AIC |
| --- | --- | --- | --- | --- |
| Model 1 | Maturity proportion ~ 1 | 1071 | 0.0 | 1486.0 |
| Model 2 | Maturity proportion ~ s (Ordinal date, *k* = 6, bs = "cc") | 1067 | 17.3 | 1375.9 |
| Model 3 | Maturity proportion ~ s (Ordinal date, by = population, *k* = 8, bs = "cc") | 1062 | 28.8 | 1282.2 |
| Model 4 | Maturity proportion ~ s (Ordinal date, by = population:sex, *k* = 8, bs = "cc") | 1052 | 36.3 | 1195.7 |
| Model 5 | Maturity proportion ~ s (Ordinal date, by = population:sex, *k* = 8, bs = "cc") + sex | 1051 | 59.4 | 722.4 |

AIC: Akaike’s information criterion.

Table S4. List of all phenotypes examined for QTL analysis. Significance levels of false discovery rate (FDR) by genome-wide permutation tests with multiple peaks (significant QTL: FDR < 0.05, suggestive QTL: 0.05 < FDR < 0.10).


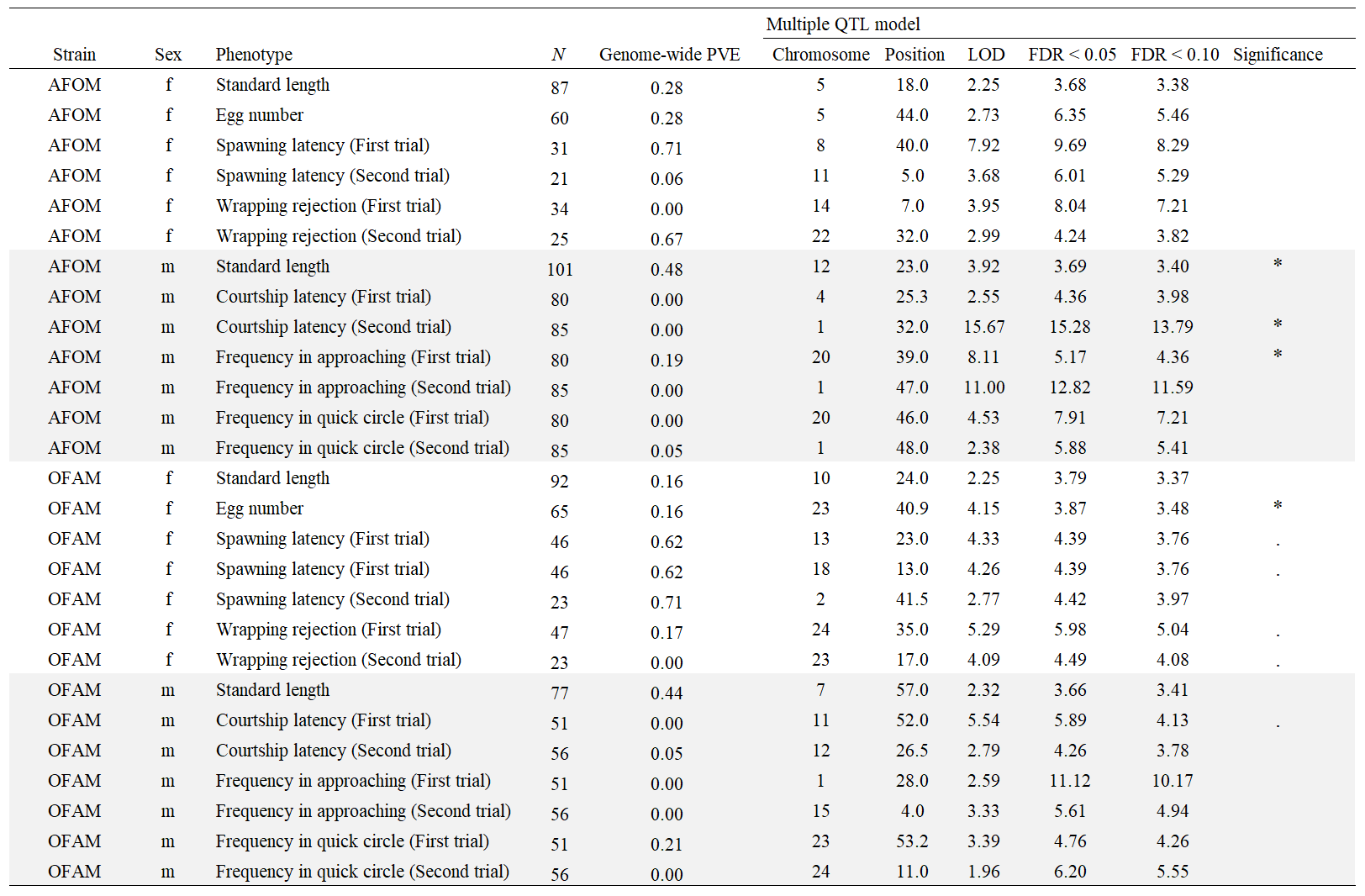


PVE: Phenotypic variance explained; LOD: Logalithm of the odds; FDR: False discovery rate; . 0.05 < FDR < 0.10; * FDR < 0.05.

Table S5. Top 10 gene ontology terms obtained from GO enrichment analysis, sorted by ascending enrichment FDR.


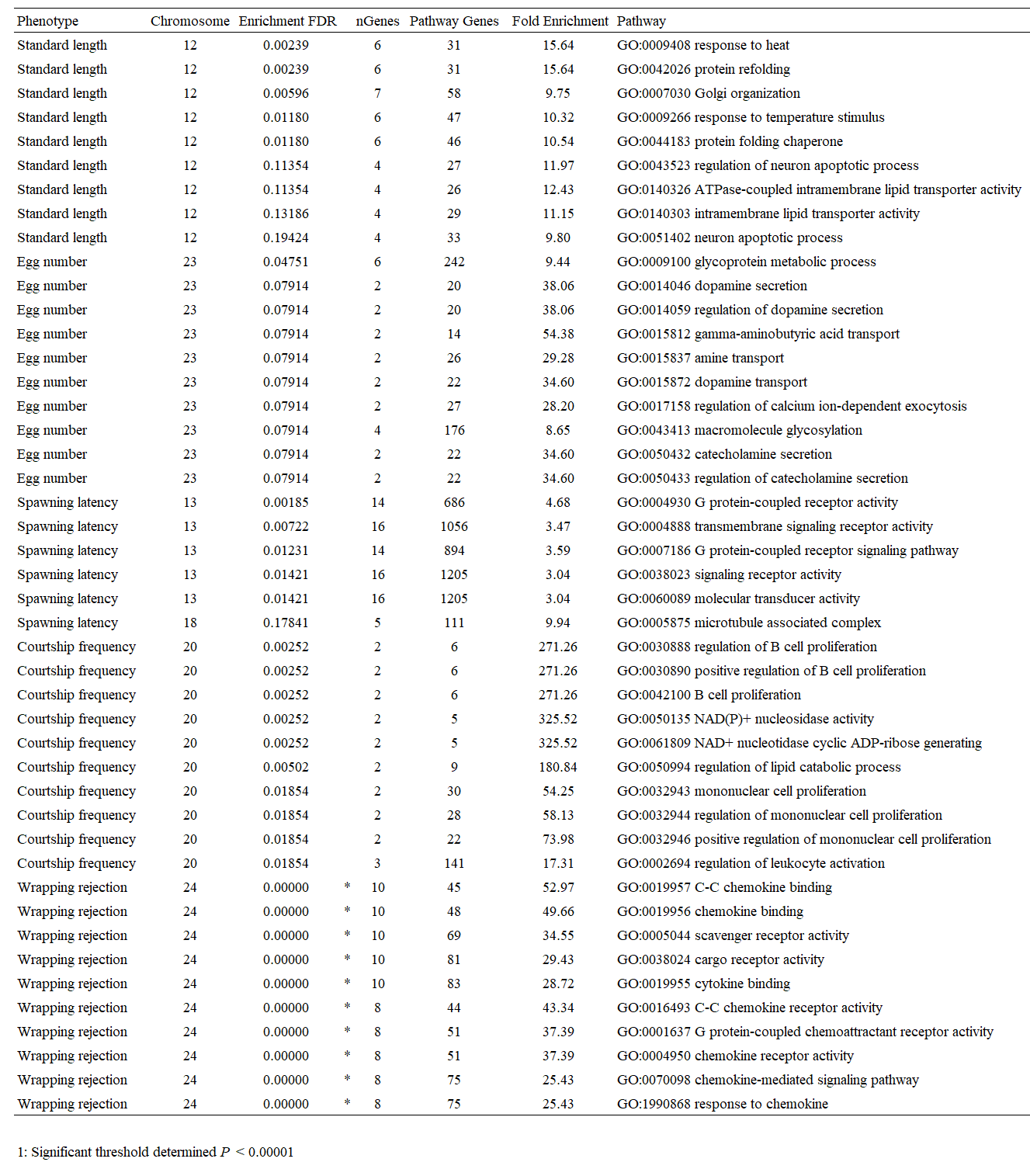


Table S6. Candidate genes involved in behavior, oogenesis, and reproduction.

**
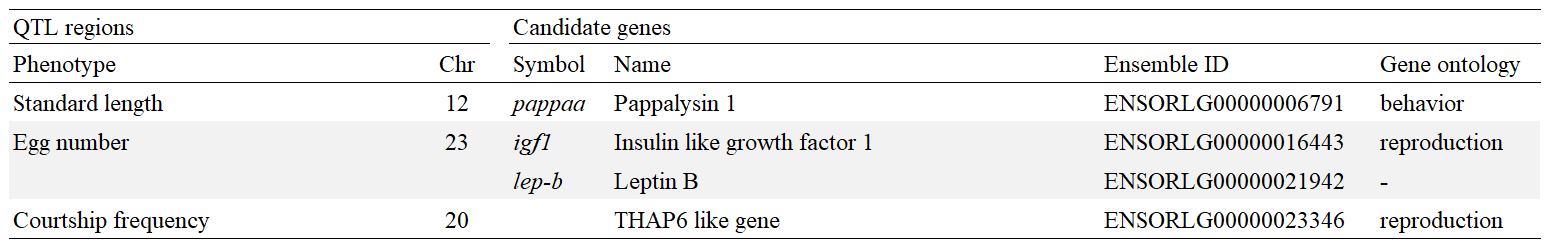
**
